# Distinct brain extracellular vesicle microRNA profiles differ in frontotemporal dementia and Alzheimer’s disease

**DOI:** 10.1093/braincomms/fcag277

**Published:** 2026-07-21

**Authors:** Joseph Morgan, Toby Aarons, Arijit Mukhopadhyay, Gemma Lace

**Affiliations:** Translational Medicine Laboratory, Biomedical Research and Innovations Centre, University of Salford, Manchester M5 4NT, United Kingdom; Translational Medicine Laboratory, Biomedical Research and Innovations Centre, University of Salford, Manchester M5 4NT, United Kingdom; Translational Medicine Laboratory, Biomedical Research and Innovations Centre, University of Salford, Manchester M5 4NT, United Kingdom; Translational Medicine Laboratory, Biomedical Research and Innovations Centre, University of Salford, Manchester M5 4NT, United Kingdom

**Keywords:** dementia, brain, extracellular vesicles, MicroRNA, biomarkers

## Abstract

Dementia is a syndrome caused by various diseases including Alzheimer's disease (AD) and frontotemporal dementia (FTD) with an estimated global prevalence of 60 million individuals. Recently, therapeutic development in the dementia field has accelerated, with the introduction of monoclonal antibody therapeutics such as Lecanemab and Donanemab. However, AD and FTD patients are still either diagnosed too late to benefit from available therapies or are misdiagnosed due to the clinical overlap between dementia subgroups making therapeutic intervention challenging. This highlights a real need to improve early diagnostic tools of neurodegenerative disease (ND) biomarkers. A potential source of such biomarkers come from small extracellular vesicles (sEVs), groups of cell-derived, lipid-bound assemblies with the capability to cross the blood–brain barrier (BBB) and known to carry pathogenic proteins associated with AD and FTD. A known cargo of sEVs is microRNA (miRNA), regulatory molecules that post-transcriptionally silence gene expression including transcripts of autophagic systems, processes which dysfunction in dementia-causing diseases leading to toxic aggregate build-up, causing neurodegeneration. The targeting of functional machineries in macroautophagy (MA) and chaperone-mediated autophagy (CMA) by different miRNA may vary between AD and FTD mutations, leading to potential biomarkers of disease being highlighted. Through isolating sEVs from the frontal cortex of post-mortem brain tissue of AD, FTD-*MAPT*, FTD-*C9orf72*, FTD-*GRN* and no-disease control patients (Manchester Brain Bank), miRNA cargoes were analysed and compared using real-time quantitative PCR (RT-qPCR). Seven autophagy-associated miRNA candidates (MA: miR-124-3p, miR-30a-5p, miR-128-3p; and CMA: miR-224-5p, miR-373-5p, miR-106a-3p and miR-26b-5p) were tested to identify dementia sub-group variations, used alongside small RNA-sequencing to explore broader miRNA variation within sEV populations. Of the miRNA tested miR-224-5p (*P* = 1.76 × 10^−5^) and miR-106a-3p (*P* = 0.033) showed significant group differences, and further significant pairwise comparison differences [miR-224-5p: AD fold change (FC) = 4.29, MAPT FC = 7.62; miR-106a-5p: AD FC = 5.59] when compared with no disease controls and other dementia subgroups, potentially showing initial diagnostic and differentiating potential. Small RNA-sequencing results revealed 8 AD, 2 FTD-*GRN*, 52 FTD-*MAPT* and 12 FTD-*C9orf72* differentially expressed sEV-miRNAs when compared with no disease controls. Further direct comparisons between AD versus FTD mutation-derived sEV cargoes, and even FTD mutation versus FTD mutation-derived sEV cargoes, identified additional miRNA with differentiating capabilities. These findings demonstrate sEV-derived miRNA signatures vary across dementia sub-types and suggest potential roles of sEV cargoes in both disease diagnostics and identifying drivers of ND, such as autophagic impairments and signalling pathways.

## Teaser

Morgan *et al*. report specific Alzheimer’s disease and frontotemporal dementia brain-derived small extracellular vesicle microRNA cargoes associated with autophagy (miR-30a-5p, miR-128-3p, miR-224-5p, miR-106a-3p) show initial diagnostic and differentiating potential compared to no disease, Alzheimer’s and frontotemporal dementia mutations. Through small RNA-sequencing, Morgan *et al*. also demonstrate foundational variations within small extracellular vesicle global microRNA environment signatures.

## Graphical Abstract

**Graphical Abstract:**
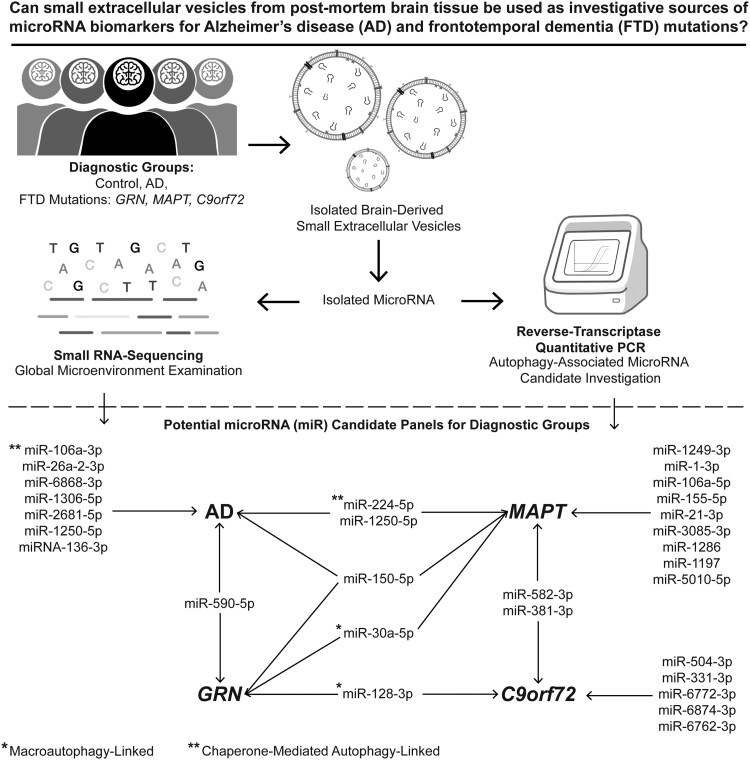

## Introduction

The term dementia describes a group of symptoms including memory loss, cognitive impairment, personality changes and communication deficits, with estimations showing around 55 million people lived with dementia globally in 2019, and projections suggesting these figures could rise to 139 million by 2050.^1^ Despite recent developments within the therapeutic field of Alzheimer’s disease (AD) through the production and consequential acceptance of monoclonal antibody treatments to global markets, diagnostics of dementia subtypes still fall short. Whilst drugs, such as Donanemab and Lecanemab, act through targeting and removing Aβ structures resulting in a decline in AD progression, there is still no precise and cost-effective way to diagnose diseases early enough for patients to benefit from such treatments.

Various diagnostic complexities exist when diagnosing and differentiating dementia-causing diseases. Firstly, alongside AD being the most common cause of dementia, frontotemporal dementia (FTD) is the second most common cause of early-onset of dementia,^2,3^ where in the latter stages of both diseases symptoms are more clinically heterogenous, yet in the earlier stages they share many overlapping clinical manifestations.^4^ Secondly, FTD mutations such as FTD-*GRN*, FTD-*MAPT* and FTD-*C9orf72* have symptomatic overlap, again furthering the challenges of early diagnosis.^5^ Thirdly, to add extra complexities, FTD shares genetic mutations, pathological phenotypes and clinical signs with that of amyotrophic lateral sclerosis (ALS),^6^ again making early differentiation of diseases intricate. Lastly, both AD and FTD share pathological commonalities; the largest being the accumulation of toxic proteins within areas of the brain impacted by neurodegeneration.^7^ In AD, the cleavage of amyloid precursor protein (APP) generates the build-up of pathogenic Aβ monomers, such as Aβ_42_, which aggregate together to form larger amyloid oligomers, fibrils and plaques.^8^ Alternatively, in FTD-*GRN* and FTD-*C9orf72*, through the haploinsufficiency of both progranulin and C9orf72 proteins, pathological TDP43 inclusions, a nuclear protein involved in the regulation of RNA, increase causing neuronal cell death.^9^ This is not the only pathological similarity however, as in AD, the build-up of amyloid species within the brain leads to increases in neuronal oxidative stress, ultimately driving the hyperphosphorylation (and other post-translational modifications) of the microtubule-associated protein tau (MAPT).^10^ Tau hyperphosphorylation results in microtubules disassociation from neuronal cytoskeletons to form neurofibrillary tangles (NFTs) which further exacerbates oxidative stress and pathogenicity within neuronal cells.^11,12^ Aggregates of hyperphosphorylated tau are also prevalent in FTD-*MAPT* cases, where the mutation alters the tau structure, driving further post-translational modifications to occur, thus neuronal cell death.^13^

Proteostatic imbalances through the failure to clear protein accumulations may be caused through specific autophagic machinery impairments, including macroautophagy (MA) components (BCl-1, p62 and LC3) or chaperone-mediated autophagy (CMA) components (Hsc70 and LAMP2A), placing autophagy in the spotlight in the dementia field.^14^ Under healthy conditions, MA and CMA function to identify waste products and facilitate their clearance from the brain.^15^ However, not only do neurotoxic aggregations further these impairments, but each of the FTD mutations are known to negatively influence autophagy pathways further evidencing the significance of these pathways in neurodegenerative disease (ND).^16-18^

Given the need to improve early disease diagnosis and understand the early physiological disease drivers to ensure patients are channelled to the most appropriate therapeutic pathways, small extracellular vesicles (sEVs) as a source of disease biomarkers have received increasing attention.^19,20^ The function of sEVs is to deliver various proteomic, transcriptomic and genomic cargoes via cell-to-cell communication, with evidence demonstrating such cargoes may provide cellular clues on brain malfunction drivers.^21^ As these cross the blood–brain–barrier to enter peripheral fluids such as blood, cerebrospinal fluid and saliva, these may provide minimally-invasive means of understanding brain changes.^22^ Understandably, earlier studies have focussed on sEV protein cargoes from peripheral fluids, particularly on known pathogenic indictors such as Aβ and tau.^19,20^ However, other cargoes such as microRNA (miRNA) are lesser-known, with few studies having explored changes in sEV cargoes from the primary site of damage—the brain. Around 18-25 nucleotides in length, miRNA play a key role in post-transcriptional gene regulation^21^ with evidence showing multiple miRNAs regulating key components of MA and CMA. Furthermore, dysregulations of numerous miRNAs are reported across neurodegenerative disorders, where in 2015, Cheng *et al*.^22^ studied 16 serum-derived miRNAs of patients, and through coupling with risk factor data, including age, sex and APOE ε allele status of a patient, AD was stated to be predicted with 87% sensitivity and 77% specificity.

Given the challenges associated with early diagnosis and differentiation between different dementia subtypes, and the need to understand early physiological pathway dysregulation to ensure appropriate early therapeutic intervention, this exploratory study aims to identify potential miRNA biomarkers of AD and the three main genetic mutation backgrounds of FTD (FTD-*GRN*, FTD-*MAPT* and FTD-*C9orf72*) from post-mortem total brain-derived sEVs (BDsEVs). Autophagic dysregulation was the focus of this exploratory study, where multiple miRNAs with links to autophagy (miR-124-3p, miR-30a-5p, miR-128-3p, miR-224-5p, miR-373-5p, miR-106a-3p and miR-26b-5p) were tested to examine alteration patterns across dementia and control groups, as a potential candidate panel of miRNA biomarkers. Global miRNA microenvironments within the sEVs were also investigated to further explore novel miRNAs that could be utilized to differentiate between different disease groups and may show promise for further exploration in peripheral fluids as a less invasive disease biomarker.

## Materials and methods

This study was completed in the Translational Medicine Laboratory at the University of Salford, where BDsEVs were isolated from post-mortem brain tissue (AD and FTD mutations), characterized and analysed using RT-qPCR and small RNA-seq. Given the limited availability of post-mortem tissue, this study was designed as an exploratory analysis to identify candidate miRNAs and pathway alterations for future validation.

### Human brain tissue

Frozen post-mortem human frontal lobe samples from AD, FTD-*GRN*, FTD-*MAPT*, FTD-*C9orf72* and no disease controls (five tissues per group) were obtained from the Manchester Brain Bank, part of the Brains for Dementia Network Initiative in the UK. The brain tissue was collected, dissected and stored using protocols established by the BrainNet Europe consortium.^23^ Each AD and FTD case were assessed for a clinical and pathological diagnosis,^24-26^ including APOE genotyping and Braak Stage evaluation (Table 1). Additionally, no disease controls had no clinical diagnosis, where pathological conditions showed age changes only.

**Table 1:** Demographic information of the frozen brain tissue samples used in the study

Sample cohort	Sample number (*n*)	Mean age ± SD	Gender		APOE Variation					
			M	F	2/2	2/3	2/4	3/3	3/4	4/4
Control	5	89.4 ± 4.4	2	3	-	1	-	4	-	-
AD	5	76.4 ± 11.2	3	2	-	-	-	2	2	1
FTD-*GRN*	5	70.6 ± 2.7	3	2	-	-	-	5	-	-
FTD-*MAPT*	5	59.4 ± 4.2	1	4	-	-	-	5	-	-
FTD-*C9orf72*	5	71.0 ± 7.0	2	3	-	-	-	5	-	-
*Total*	*25*	*73.3* ± *4.9*	*11*	*14*	*-*	*1*	*-*	*21*	*2*	*1*

Information includes sample number (±SE), average age (of death), gender split ration (male:female) and the APOE profile of case sample.

### Ethics statement

Ethical approval was obtained from the Manchester brain bank for the storage and research use of fresh-frozen human brain tissue samples (REC reference 09/H0906/52), with local ethical approval for this project granted by the University of Salford ethics board (reference 0754).

### Isolation of brain-derived small extracellular vesicles

Full methodology for the isolation of total BDsEVs was previously described in Morgan *et al*.^27^ Firstly, 250 mg frozen brain tissue was sliced (no disease control, AD, FTD mutation samples) and placed in 2 mL 75 U/mL collagenase type-III/Hibernate-E solution per brain tissue sample. Samples incubated at 37°C for 20 min where at the 5 min interval, samples were gently inverted twice and at 10 min interval, samples were gently pipetted up and down twice using a 10 mL stripette. Samples were returned to ice with 20 μL 100× Halt™ protease inhibitors (ThermoFisher) and PhosSTOP inhibitors (Roche) and then centrifuged at 2000 × *g* for 15 min at 4°C. The cell-free supernatant samples were slowly filtered using a 0.22 μm filter, then further centrifuged at 10 000 × *g* for 48 min (4°C), where Exo-Spin™ buffer (Cell Guidance Systems) was added to the supernatant at a ratio of 2:1 and incubated overnight (4°C). The solutions were centrifuged at 10 000 × *g* for 96 min (4°C), where the pellet was resuspended in 100 μL extracellular vesicle free PBS, and the resuspension was added to an ExoSpin™ column (Cell Guidance Systems) and left to enter the column through gravity. To the column, 180 μL PBS was added where the column was centrifuged at 50 × *g* for 1 min, eluting the BDsEVs. Isolation workflow summarized in Supplementary Fig. 1.

### Characterization of brain-derived small extracellular vesicles

In accordance with the minimal information for studies of extracellular vesicles (MISEV) guidelines,^28,29^ three methods were used to characterize and validate the isolated sEVs: nanoparticle tracking analysis (NTA)/fluorescent NTA, western blotting and transmission electron microscopy (TEM), as described.^27^ Further details on full NTA, western blot, and TEM methodologies can be found in Supplementary Methodology 1, with western blot antibody concentrations and dilutions in Supplementary Table 1 and NTA run settings in Supplementary Table 5.

### Isolation of total RNA population of brain-derived small extracellular vesicles

Prior to total RNA isolation, a preparative step of treating each BDsEV sample with proteinase K and RNase A was performed to ensure all downstream isolated RNA cargoes came strictly from within the BDsEVs themselves. To 50 μL of sEV sample, 1 μL 20 μg/μL proteinase K (ThermoFisher) was added and incubated for 37°C for 30 min, where any reaction was stopped using 0.5 μL 100× Halt™ and PhosSTOP™, and incubating on ice for 10 min. To each sample, 1 μL 10 μg/μL RNase A was added and incubated at 37°C for 30 min, ready for RNA isolation. Total RNA isolation was performed using a RNeasy RNA isolation kit (Qiagen). Samples were tested against untreated EV samples (untreated from proteinase K/RNase A) to confirm only external RNAs were degraded and internal RNAs remained protected (as shown in Morgan *et al*.^27^). To quantity the amount of miRNA within each sample, a Qubit™ microRNA Assay Kits were used and read on a Qubit™ 3 Fluorometer (miRNA assay setting).

### Reverse-transcriptase quantitative PCR

Autophagy-linked candidate miRNAs were selected based on literature evidence showing connections to autophagy-related pathways and ND mechanisms.^30-34^ Selection criteria included: prior association with autophagy regulation; and reported dysregulation in ND conditions. This approach was designed to test biologically informed hypothesis rather than screen for differential expression.

To analyse specific miRNA candidates, reverse-transcriptase quantitative PCR (RT-qPCR) was performed. To generate cDNA for RT-qPCR, a QuantiMiR RT Small RNA Quantification System Kit was used. To a thin-walled PCR tube, 5 µL 10 ng total RNA, 2 µL 5× PolyA Buffer, 1.5 µL 5 mM ATP, 1 µL 25 mM MnCl_2_, 1 µL spike-in cel-miR-39 solution (8 × 10^9^ copies/mL) and 0.5 µL PolyA polymerase was added and incubated in a thermocycler at 37°C for 30 min. After incubation, to each sample, 0.5 µL oligo dT adapter was added and incubated at 60°C for 5 min, whereafter, the samples were left to cool at room temperature for 2 min. A MasterMix containing 4 μL 5× RT buffer, 2 μL dNTP mix, 1.5 μL 0.1 M DTT, 1 μL reverse transcriptase and 0.5 μL RNase-free H_2_O was made, where 9 μL total Master Mix was added to each sample. The samples were incubated at 42°C for 1 h (lid temperature 105°C) and was subsequently heated at 95°C for 10 min. The cDNA produced was diluted 1:10 and was ready for RT-qPCR.

For RT-qPCR, per reaction, 15 μL 2× SYBR Green RT-qPCR MasterMix buffer (Meridian Bioscience), 1 μL 10 μM universal reverse primer (System Bioscience), 1 μL 10 μM miRNA target-specific forward primer (Eurofins), 12.5 μL RNase-free water (Fisher Scientific) and 1 μL 10 ng cDNA was added and mixed. The forward miRNA primer sequences (Supplementary Table 2) were obtained through the miRbase (https://www.mirbase.org/). Cycling conditions found in Supplementary Table 3. The delta–delta Ct (2^−ΔΔCt^) method was used to calculate the relative fold gene expression of the samples, compared with that of a spiked-in cel-mir-39 reference, with 2 technical replicates performed for each sample per experiment.

### Small RNA-sequencing

Small RNA-sequencing (small RNA-seq) was used as an exploratory genome-wide method to investigate the entire microRNA cargo within BDsEV environments. BDsEVs. The library was prepared using the small RNA-seq library preparation kit (Lexogen), where both 3′ and 5′ adapters were added, alongside P5 and P7 (with indices), and amplified using endpoint PCR (Supplementary Table 4). Full details of methodologies are found in Supplementary Methodology 2. Multiplexed libraries were run on the Illumina MiSeq instrument using 50-cycle base pair reads. Bioinformatic analysis workflow can be found in Supplementary Methodology 3.

### Statistical analysis

Statistical analyses were performed to analyse the differences in mean age, size distributions of BDsEVs of each diagnostic group, differences in CD marker concentration and differences in miRNA expression using RT-qPCR and small RNA-seq, all being performed in R (v4.5.2) (in R Studio v2025.09.2).

For age difference analysis, a one-way ANOVA was performed to identify age differences between diagnostic groups. Where significant effects were observed, post hoc pairwise comparisons using Tukey’s honestly significant difference was used to account for multiple comparisons, where significance was *P* < 0.05.

For BDsEV size distribution analysis, area under the curve (AUC) was calculated for each replicate across predefined size windows: EV-enriched (30–200 nm), mid-range (150–300 nm) and full measured range (0–900 nm); whereafter non-parametric tests were applied. Group differences were assessed using Kruskal–Wallis rank-sum tests, where further pairwise comparisons between groups were performed using Wilcoxon-rank-sum tests and corrections for multiple comparisons (Benjamini–Hochberg false discovery rate (FDR) correction). Adjusted *P* values (*P* adj) of <0.05 were considered statistically significant. Additionally, the difference in CD marker variation between groups was tested using one-way ANOVA (significance of *P* < 0.05).

For RT-qPCR analysis, group differences in BDsEV miRNA expression were tested using one-way ANOVAs. Where applicable, post-hoc pairwise comparisons were performed using two-sample *t*-tests and corrections for multiple comparisons (Holm corrections).

For differential expression analysis, DESeq 2 (v1.50.2) analysis was used where count data were normalized using the median-of ratios method, and dispersion estimates were obtained using DESeq2 empirical Bayes shrinkage. Wald tests were used to evaluate contrasts between diagnostic groups (disease versus control; AD versus FTD mutation; FTD mutation versus FTD mutation) with corrections for multiple comparisons (Benjamini–Hochberg FDR). Due to the small sample size and inherently low abundance of many miRNAs, no transcripts survived FDR correction; therefore, nominal *P* values (*P* < 0.05) were used to identify exploratory candidate biomarkers. Volcano plots were generated using ggplot2, displaying log2 fold change versus −log_10_(*P* value), with both up and downregulated miRNAs annotated. Pathway enrichment analyses were conducted using KEGG pathway annotation via clusterProfiler R package, with pathway information retrieved dynamically from the KEGG database (accessed December 2025) (Supplementary Data 5; Supplementary Fig. 4). Principal component analysis (PCA) visualizations of variance-stabilized counts were generated for exploratory assessment of sample structure (Supplementary Fig. 5).

## Results

### Age demographic analysis

Using one-way ANOVA, significant differences in mean ages were shown (Table 1: *F*(4,20) = 13.56, *P* < 0.001). Post hoc Tukey analysis showed that the control group was significantly older than all disease subgroups (AD *P* = 0.039; FTD-*GRN P* = 0.001; FTD-*MAPT P* = <0.001; FTD-*C9orf72 P* = 0.002) and FTD-MAPT is significantly younger than AD (*P* = 0.004) (Supplementary Data 1).

### Characterization of isolated brain-derived small extracellular vesicles

Three methods were used to characterize the isolated sEVs in accordance with MISEV guidelines: NTA/fNTA, western blotting and TEM.^28,29^

**Figure 1:**
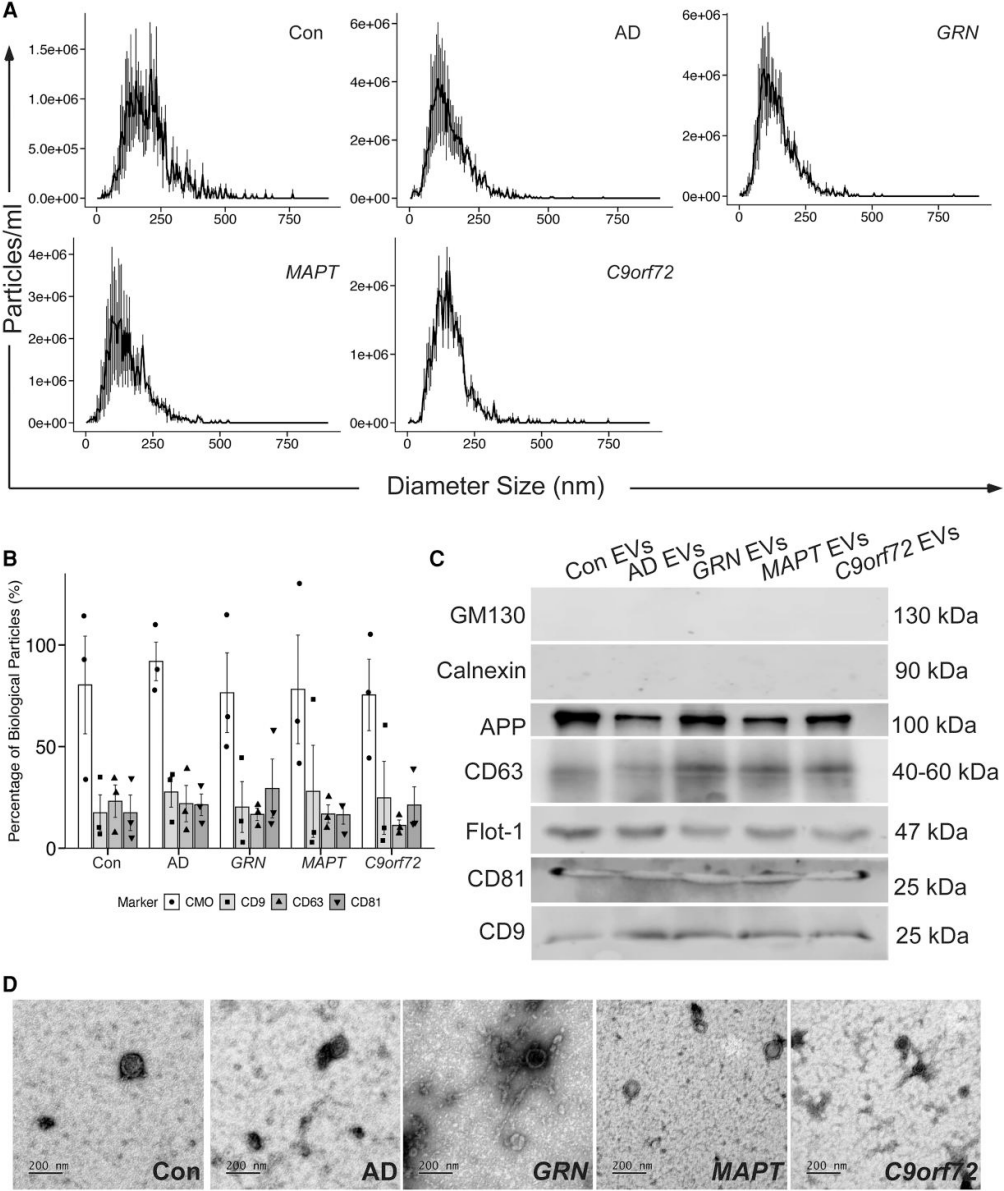
**Characterization of BDsEVs.** (**A**) Using fNTA, the average particle size distributions of CMO-stained biological BDsEVs within diagnostic groups (no disease control (Con), AD, FTD-*GRN*, FTD-*MAPT*, FTD-*C9orf72*) falling in the range of 0–902.5 nm (±SEM error bars, sample size *n* = 3). No significant differences in overall group level (Kruskal–Wallis) and individual groups (Wilcoxon with Benjamini–Hochberg FDR corrections) were observed (*P* = 0.117, *F* = 1.938). (**B**) Analysis using fluorescent nanoparticle tracking analysis (fNTA) shows the percentage of CD-positive particles within the biological particles populations (each bar represents the mean with ± SEM error bars, where individual plot represents data from individual brain tissue samples (sample size *n* = 3): circle = CMO; square = CD9; triangle = CD63, inverted triangle = CD81). No significant differences in overall group level of CD markers between diagnostic groups (One Way ANOVA: CD9 *P* = 0.99 *F* = 0.06; CD63 *P* = 0.42 *F* = 1.08; CD81 *P* = 0.80, *F* = 0.40). (**C**) Western blot analysis shows the presence of neuronal-specific APP, hallmark sEV membrane (CD9, CD63 and CD81) and cytosolic proteins (Flot-1) in BDsEVs, and the absence of cell-derived calnexin and Golgi-derived GM130. (**D**) TEM images of no disease control, AD, FTD-*GRN*, FTD-*MAPT* and FTD-*C9orf72* sEV-like structures within the samples (size bar = 200 nm). CMO, cell mask orange; EVs, extracellular vesicles.

Using fNTA, the size range of most particles fell within the characteristic range of sEVs (50–200 nm, Fig. 1A) as defined by MISEV guidelines, where whilst NTA revealed largely conserved median particle diameters (D50), the size distribution spread between groups visually indicated potential differences. To measure differences, AUC was calculated across predefined size ranges, where no significance in size distribution differences was detected across any size range following Kruskal–Wallis testing and Wilcoxon rank-sum tests with Benjamini–Hochberg-corrected pairwise comparisons (adj *P* > 0.05, Supplementary Data 2). Native size distribution plots are found in Supplementary Fig. 2, and further information regarding average CMO EV counts in Supplementary Table 5.

Using NTA, the concentration of total particles in each sample obtained after sEV isolation fell between 1.25 × 10^11^ and 1.37 × 10^11^ particle/mL, where >75% of all particles isolated contained a biological membrane stained by the CMO dye (Fig. 1B). Through further probing, tetraspanin-positive particles are shown present within the biological particle subpopulation at different concentrations, where the assumption that all particles may bear one or more tetraspanin on the surface. Of biological particles in no disease samples, 17.41% were CD9-positive, 23.11% were CD-63 positive and 17.45% were CD81-positive, in comparison to AD samples, where 27.62% of biological particles were CD9-positive, 21.94% were CD63-positive and 21.37% were CD81-positive. In FTD mutation samples, FTD-*GRN* biological particles contained 20.27% CD9-positive, 16.63% CD63-positive and 29.37% CD81-positive particles; FTD-*MAPT* biological particles contained 29.21% CD9-positive, 16.82% CD63-positive and 16.43% CD81-positive particles; and FTD-*C9orf72* biological particles contained 24.79% CD9-positive, 11.21% CD63-positive and 21.22% CD81-positive particles (Fig. 1B). After using a one-way ANOVA to test group differences between CD markers within each diagnostic group, no significance was found (CD9 *P* = 0.99; CD63 *P* = 0.42; CD81 *P* = 0.80) (Supplementary Data 2).

Western blotting was used to show the presence of characteristic membrane and cytosolic proteins found within sEVs from past research, alongside the absence of cell-derived markers (Fig. 1C). Using western blotting, 5 positive markers of sEVs were probed (CD9, CD63, CD81, Flot-1 and neuronal-specific APP), alongside two negative markers (Calnexin and GM130), where the BDsEV samples from each disease were observed against whole BH samples. In all samples (no disease control, AD and FTD), positive staining showed the presence of all tetraspanins and flot-1 in BH and BDsEV samples, with thicker bands shown in BH samples compared with BDsEVs. Furthermore, only within BH samples, ER-associated calnexin and Golgi-derived GM130 are present, with no presence shown markers within BDsEVs, and the absence of any cell-derived protein markers, suggest minimal cellular compartment contamination and a refined isolated sample (full blot images in Supplementary Fig. 3).

Lastly, TEM was used to visualize the morphology and relative size of the isolated BDsEVs (Fig. 1D). In each disease sample, small membrane-bound circular particles are shown through the presence of darker staining that generally fall below 200 nm.

### Autophagy-associated candidate MicroRNAs are differentially expressed between AD and FTD

**Figure 2:**
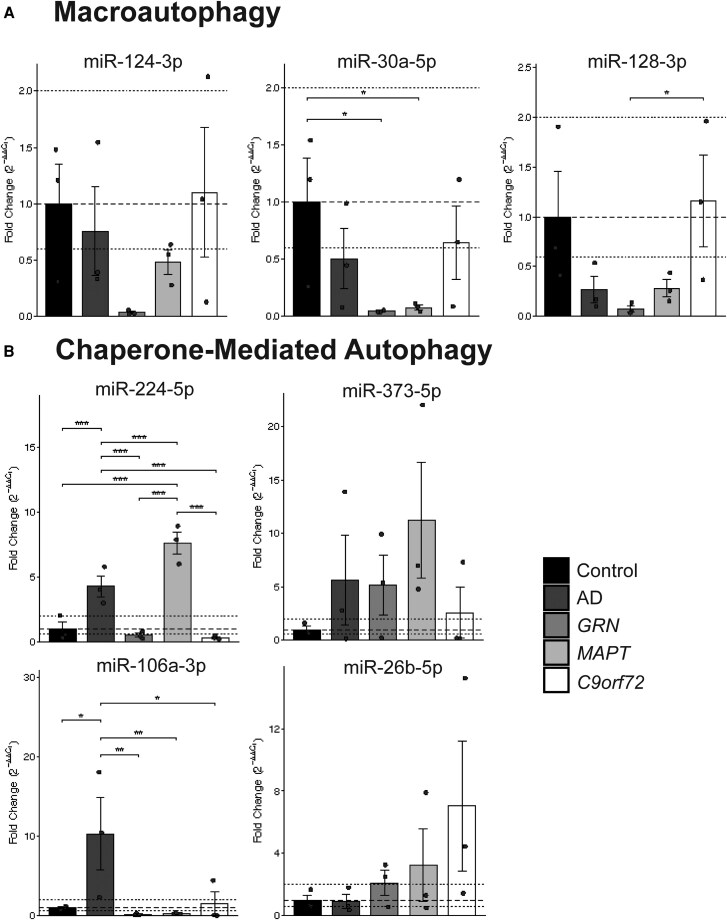
**Variation in autophagy-associated BDsEV miRNA cargoes.** Isolated BDsEV (**A**) MA-associated miR-124-3p, miR-30a-5p, miR-128-3p and (**B**) CMA-associated miR-224-5p, miR-373-5p, miR-106a-3p and miR-26b-5p regulation patterns within diagnostic groups. FCs were normalized against cel-mir-39 spike-in and referenced against no disease sample FCs (plotted). Each bar represents the mean (±SEM), where each individual plot represents data from individual brain tissue samples (sample size *n* = 3) with 3 dotted lines representing fold change of 0.6 (downregulation), 2 (upregulation) and 1. One way ANOVA was performed to identify significant group differences, whereafter pairwise comparison unadjusted significant *P* values are plotted (*P < 0.05, **P < 0.01, ***P < 0.001). From the One-Way ANOVA, only miR-224-5p (*P* = 1.76 × 10^−5^, *F* = 29.028) and miR-106a-3p (*P* = 0.033, *F* = 4.061) showed significant differences between groups. Significant differences detected using pairwise comparisons were observed in miR-30a-5p (Con versus FTD-*GRN*: *P* = 0.0232; Con versus FTD-*MAPT*: *P* = 0.0273), miR-128-3p (FTD-*GRN* versus FTD-*C9orf72*: *P* = 0.0278), miR-224-5p (Con versus AD: *P* = 0.0026; Con versus FTD-*MAPT*: *P* = 1.18 × 10^−5^; AD versus FTD-*GRN*: *P* = 0.0011; AD versus FTD-*MAPT*: *P* = 0.0024; AD versus FTD-*C9orf72*: *P* = 0.0008; FTD-*MAPT* versus FTD-*GRN*: 6.45 × 10^−6^; FTD-*MAPT* versus FTD-*C9orf72*: 5.22 × 10^−6^) and miR-106a-3p (Con versus AD: *P* = 0.0117; AD versus FTD-*GRN*: *P* = 0.0073; AD versus FTD-*MAPT*: *P* = 0.0078; AD versus FTD-*C9orf72*: *P* = 0.0159). After Holm correction, only miR-224-5p comparisons (Con versus AD: *P* adj = 0.012; Con versus FTD-*MAPT*: *P* adj = 9.41 × 10^−5^; AD versus FTD-*GRN* = *P* adj = 0.0065; AD versus FTD-*MAPT*: *P* adj = 0.0120; AD versus FTD-*C9orf72*: *P* adj = 0.0055; FTD-*MAPT* versus FTD-*GRN*: *P* adj = 5.81 × 10^−5^; FTD-*MAPT* versus FTD-*C9orf72*: *P* adj = 5.22 × 10^−5^) were significant within tauopathies.ALT TEXT: Bar charts displaying real-time quantitative PCR results of 3 macroautophagy-associated microRNAs within A, and 4 chaperone-mediated autophagy-associated microRNAs within B, with microRNA-30a-5p, microRNA-128-3p, microRNA-224-5p and microRNA-106a-3p demonstrating significant differences between Alzheimer's disease and specific frontotemporal dementia genetic subtypes.

Seven miRNA candidates (miR-124-3p, miR-30a-5p, miR-128-3p, miR-224-5p, miR-373-5p, miR-106a-3p and miR-26b-5p) identified previously within the literature were tested using RT-qPCR, where a fold change (FC) of ≥2 was considered upregulated, and a FC of ≤0.6 was considered downregulated. Raw *P* value significances were plotted in Fig. 2, and *P* adj after Holm correction will be commented upon. Fold changes and results of statistical tests (one-way ANOVA and post-hoc pairwise comparisons with Holm correction) of each miRNA of each miRNA are shown in Supplementary Data 3.

For the three MA-associated miRNA (Fig. 2A), one-way ANOVA showed no significance between group differences (miR-124-3p *P* = 0.284; miR-30a-5p *P* = 0.106; miR-128-3p *P* = 0.095). Whilst miR-124-3p showed no significant pairwise comparisons (*P* > 0.05), miR-30a-5p was showed to be significantly downregulated (FC < 0.6) in FTD-*GRN* (*P* = 0.0232) and FTD-*MAPT* (*P* = 0.0273) compared with control, yet no significance was observed after Holm correction (FTD-*GRN* and FTD-*MAPT P* adj > 0.05). Additionally, whilst miR-128-3p signature of FTD-*C9orf72* showed no significant upregulation when compared with control (*P* > 0.05), when compared with FTD-*GRN*, this showed a significant difference (*P* = 0.0278), yet this significance did not survive Holm correction (*P* adj = 0.278).

Alternatively, when testing for group differences in the CMA-associated miRNA (Fig. 2B), both miR-224-5p and miR-106a-3p showed significant differences (miR-224-5p: *F* = 29.028, *P* = 1.76 × 10^−5^; miR-106a-3p: *F* = 4.061, *P* = 0.033), where miR-373-5p and miR-26b-5p showed no significant differences (*P* > 0.05). Further pairwise comparison and Holm correction shows that in both tauopathies, miR-224-5p was significantly upregulated in comparison to no disease controls (AD *P* = 0.00264, *P* adj = 0.012; FTD-*MAPT P* = 1.18 × 10^−5^, *P* adj = 9.41 × 10^−5^) and compared with other each other and additional dementia subgroups (AD versus FTD-*GRN P* = 0.0011, *P* adj = 0.0065; AD versus FTD-*MAPT P* = 0.0024, *P* adj = 0.012; AD versus FTD-*C9orf72 P* = 0.0008, *P* adj = 0.0055; FTD-*MAPT* versus FTD-*GRN P* = 6.45 × 10^−6^, *P* adj = 5.81 × 10^−5^, FTD-*MAPT* versus FTD-*C9orf72 P* = 5.22 × 10^−6^, *P* adj = 5.22 × 10^−5^). Furthermore, miR-106a-3p was significantly upregulated in AD when compared with control (*P* = 0.0117), FTD-*GRN* (*P* = 0.0073), FTD-*MAPT* (*P* = 0.0078) and FTD-*C9orf72* (*P* = 0.0159), however these were not found to be significant after Holm correction (*P* adj >0.05). Both miR-373-5p and miR-26b-5p showed no significant differences in pairwise comparisons (*P* > 0.05).

### Genome-wide sEV microRNA profiling for brain-derived small extracellular vesicles

Small-RNA-sequencing was used to identify miRNA biomarker profiles within the microenvironments of BDsEVs from dementia subgroups. Given the small sample size, this analysis was designed as an exploratory approach, where whilst no miRNA survived FDR corrections for multiple testing (*P* adj > 0.05), nominally significant miRNAs (*P* < 0.05) were examined to identify potential biomarker candidates. Sequence run details regarding read depth, coverage and length, alongside raw counts of every miRNA (significantly differentially expressed and no expressed miRNA) can be found in Supplementary Data 4.

**Figure 3:**
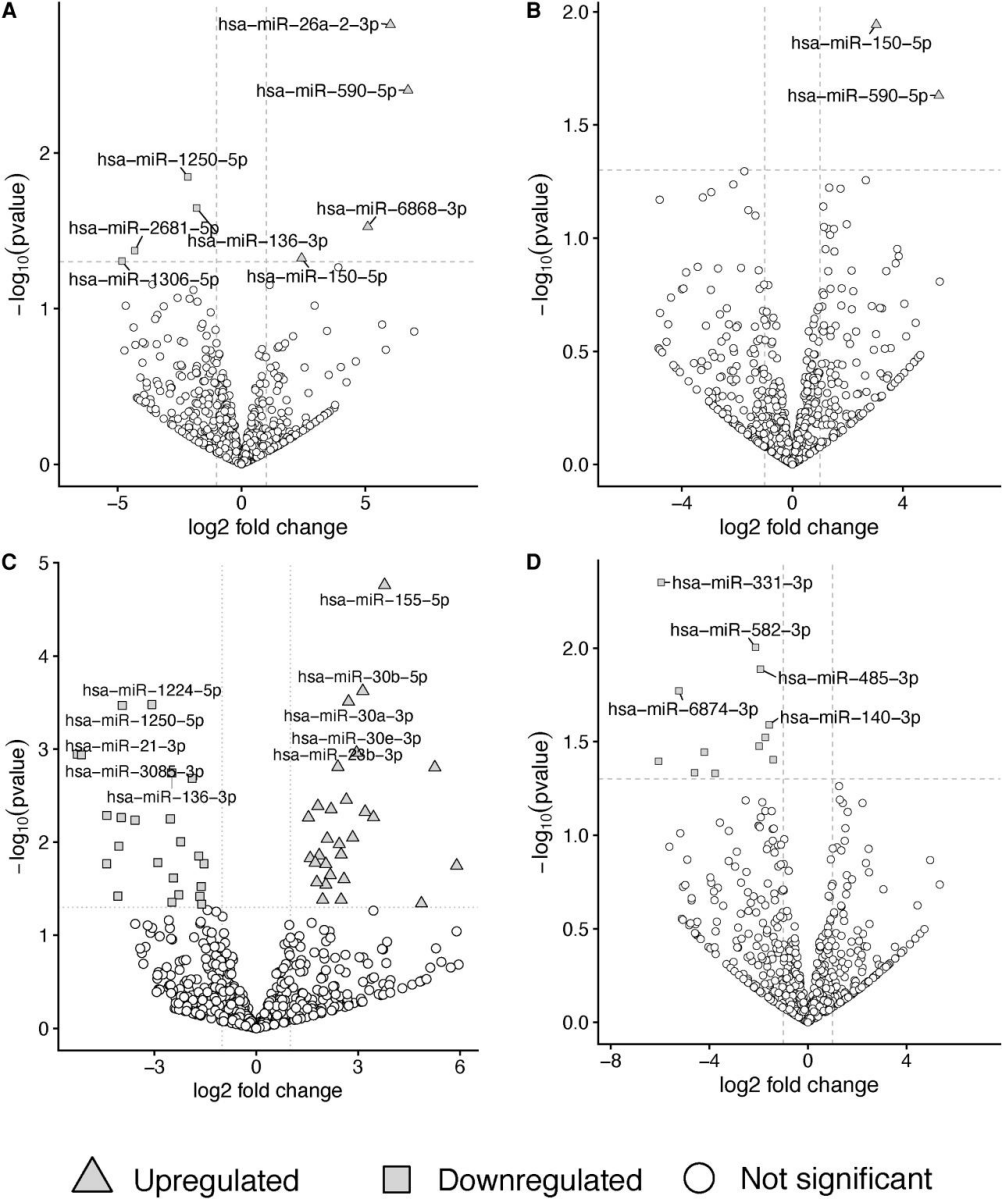
**BDsEV miRNA cargo microenvironment variation compared with no disease control samples.** Volcano plots displaying the differential expression of (**A**) AD, (**B**) FTD-*GRN*, (**C**) FTD-*MAPT* and (**D**) FTD-*C9orf72* BDsEV human (hsa) miRNA cargoes compared with no disease samples with log2FC plotted against log10*p* values. DESeq2 was ran in R which applies a negative binomial generalized linear model with Wald testing (sample size *n* = 3, biological replicates plotted). Nominally significant upregulated (triangle plot), downregulated (square plot) and not significant (circular) miRNAs are plotted, as no miRNAs survived FDR testing (with the top 5 up and downregulated miRNA labelled). Dashed lines represent log2FC of 1 and −1 and a −log10p of 1.3 (*P* < 0.05).

After performing differential expression analysis comparing AD sEV microenvironments against no disease controls, 8 miRNAs were identified (Fig. 3A), where the 4 upregulated miRNAs revealed (from highest fold change down) were miR-26a-2-3p, miR-590-5p, miR-6868-3p and miR-150-5p. The 4 downregulated (from the lowest fold change up) were miR-1306-5p, miR-2681-5p, miR-1250-5p and miR-136-3p.

As for FTD-*GRN* BDsEV miRNA environments, only 2 upregulated miRNAs (miR-590-5p and miR-150-5p) were identified when compared with no disease controls (Fig. 3B), with no downregulated miRNA shown. Following differential expression analysis on FTD-*MAPT* samples compared with no disease controls, 28 significantly upregulated miRNAs were revealed, compared with 23 downregulated miRNAs (total of 51 differentially expressed miRNAs). The top 5 upregulated miRNAs within the FTD-*MAPT* BDsEV samples were miR-1249-3p, miR-1-3p, miR-106a-5p, miR-155-5p and miR-150-5p, whereas the top 5 downregulated miRNA cargoes were miR-21-3p, miR-3085-3p, miR-1286, miR-1197 and miR-5010-5p (Fig. 3C). For FTD-*C9orf72* sEV microenvironments when compared with no disease controls, a total of 12 downregulated miRNA was identified, with no upregulated miRNA found (Fig. 3D). The top 5 downregulated miRNAs were miR-504-3p, miR-331-3p, miR-6772-3p, miR-6874-3p, miR-6762-3p.

Variations in miRNA profiles are visualized in Supplementary Fig. 6, where singular miRNAs that show differences between diseases (when compared with no disease controls) may show greater differentiating power. Examples of shared miRNAs include miR-1250-5p (downregulated in both tauopathies), or miR-136-3p (downregulated in AD, FTD-*MAPT* and FTD-*C9orf72*). Through running KEGG pathway enrichment, various pathways were identified to be targeted by the differentially expressed miRNA when compared with no disease controls (Supplementary Data 5; Supplementary Fig. 4).

### Differential expression of microRNA in AD versus different FTD mutational backgrounds

To further differentiate between distinguishing microRNA biomarkers between AD and the FTD mutational backgrounds, differential expression analysis was performed. Again, no miRNA survived correction for multiple testing (*P* adj > 0.05), therefore nomically significant miRNAs (*P* < 0.05) were examined to identify potential biomarkers for differentiation of AD against FTD mutations. Raw counts of all miRNAs for each comparison are shown in Supplementary Data 4.

**Figure 4:**
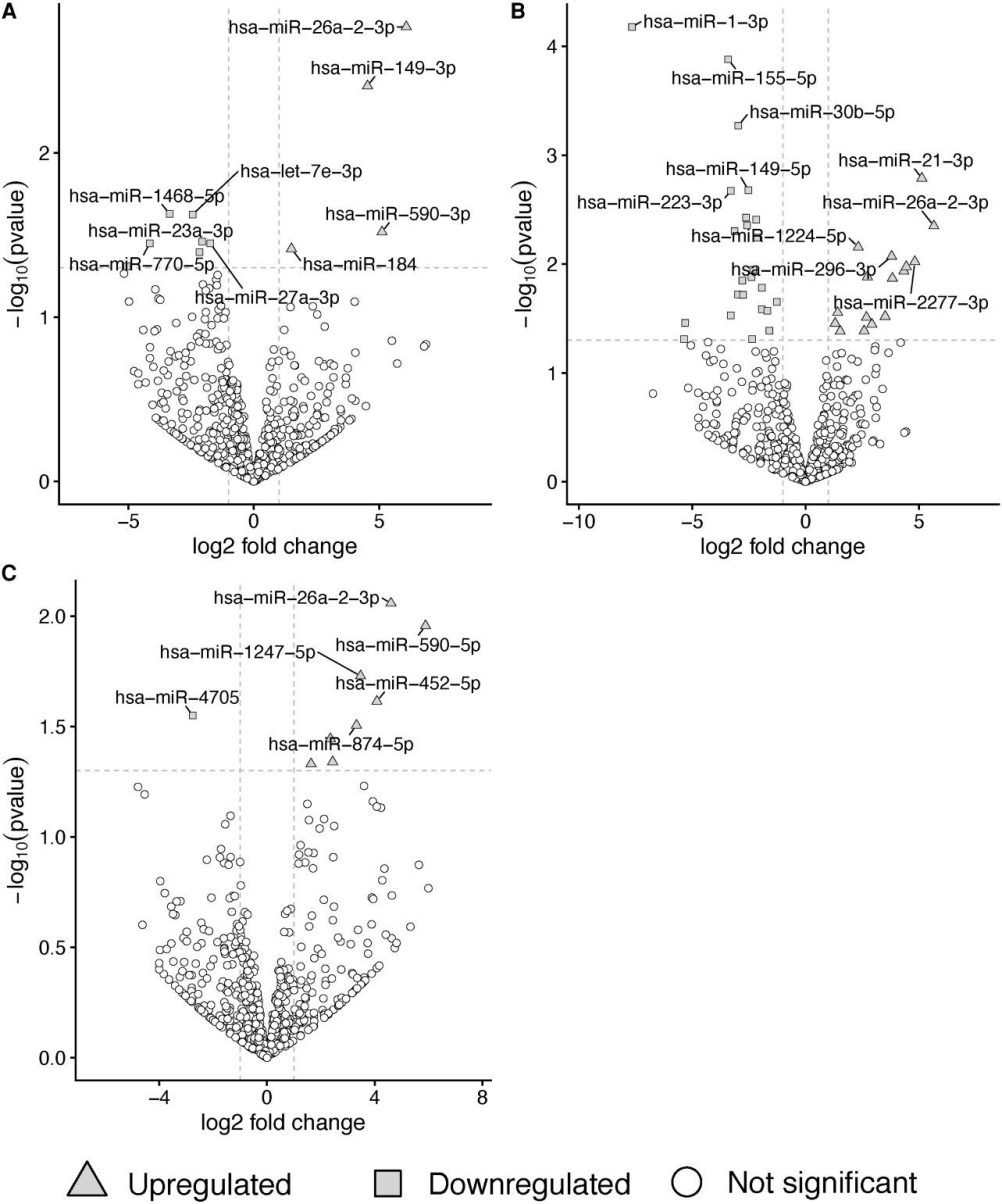
**Microenvironment variation of AD BDsEV miRNA cargoes compared with FTD mutation samples.** Volcano plots displaying the differentially expressed human (hsa) miRNA in AD BDsEVs compared with (**A**) FTD-*GRN*, (**B**) FTD-*MAPT* and (**C**) FTD-*C9orf72* BDsEV cargoes, with log2FC plotted against log10*p* values. DESeq2 was ran in R which applies a negative binomial generalized linear model with Wald testing (sample size *n* = 3, biological replicates plotted). Nominally significant upregulated (triangle plot), downregulated (square plot) and not significant (circular) miRNAs are plotted, as no miRNAs survived FDR testing (with the top 5 up and downregulated miRNA labelled). Dashed lines represent log2FC of 1 and −1 and a −log10p of 1.3 (*P* < 0.05).

When comparing AD BDsEVs miRNA microenvironments against FTD-*GRN* samples, 11 differentially expressed miRNAs were identified. The 4 upregulated miRNAs were miR-26a-2-3p, miR-590-3p, miR-149-3p and miR-184, whereas the top 5 downregulated miRNAs were miR-26a-5p, miR-770-5p, miR-1468-5p, let-7e-3p and miR-505-3p (Fig. 4A). When comparing AD to FTD-*MAPT* samples, 28 miRNAs were identified (16 upregulated, 12 downregulated), where the top five miRNAs revealed were miR-26a-2-3p, miR-21-3p, miR-2277-3p, miR-149-3p and miR-1247-5p. Alternatively, the top 5 downregulated miRNAs were miR-1-3p, miR-301b-3p, miR-20b-5p, miR-155-5p and miR-379-3p (Fig. 4B). Lastly, after comparison of AD sEV miRNA cargoes against FTD-*C9orf72* sEV miRNAs, 9 miRNAs were differentially expressed (8 upregulated versus 1 downregulated). The top 5 upregulated miRNAs identified were miR-590-5p, miR-26a-2-3p, miR-452-5p, miR-1247-5p and miR-874-5p, whereas the only downregulated found was miR-4705 (Fig. 4C).

When collating data of AD versus FTD mutation samples, specific biomarkers where the regulation patterns are not shared can be identified (Supplementary Fig. 7). Interestingly, no downregulated miRNA was common within sEV from AD samples when compared with sEV from FTD samples with different mutational backgrounds. However, miR-149-3p is upregulated in AD when compared with both FTD-*GRN* and FTD-*MAPT* samples and miR-1247-5p is upregulated in AD when compared with FTD-*GRN* and FTD-*C9orf72* sEV samples. Remarkably, when compared with all FTD samples, miR-26a-2-3p is upregulated in AD samples, a similar observation when comparing AD to no disease control samples, potentially making miR-26a-2-3p a strong candidate for further testing.

### Distinct miRNA signatures for different backgrounds of FTD

Further miRNA cargo signatures of specific FTD mutations were also identified to compare mutation against other mutations. Much like the other comparisons prior, no miRNA survived correction for multiple testing (*P* adj > 0.05), hence nomically significant miRNAs (*P* < 0.05) were examined to identify potential biomarkers for differentiation of the FTD mutations. Raw counts of all miRNAs for each comparison are shown in Supplementary Data 4.

**Figure 5:**
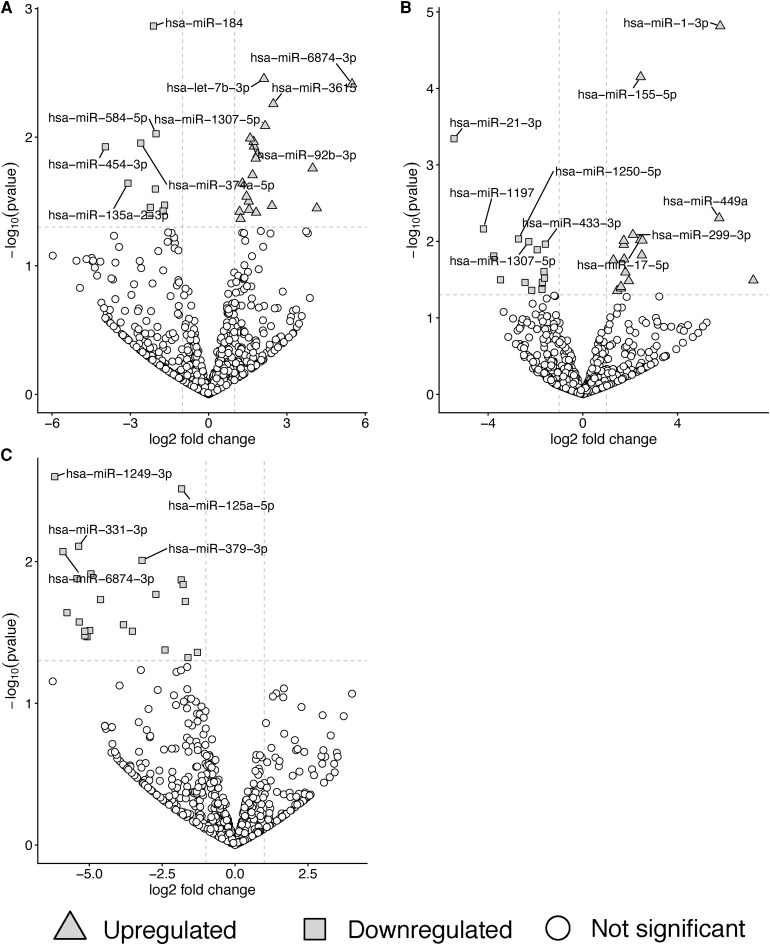
**FTD mutation BDsEV miRNA cargo microenvironment variation compared with other FTD mutation samples.** Volcano plots displaying the human (hsa) miRNA in all FTD samples after differential expression, where each graph shows (**A**) FTD-*GRN* versus FTD-*MAPT* and FTD-*C9orf72*, (**B**) FTD-*MAPT* versus FTD-*GRN* and FTD-*C9orf72* and (**C**) FTD-*C9orf72* versus FTD-*GRN* and FTD-*MAPT*, with log2FC plotted against log10*p* values. DESeq2 was ran in R which applies a negative binomial generalized linear model with Wald testing (sample size *n* = 3, biological replicates plotted). Nominally significant upregulated (triangle plot), downregulated (square plot) and not significant (circular) miRNAs are plotted, as no miRNAs survived FDR testing (with the top 5 up and downregulated miRNA labelled). Dashed lines represent log2FC of 1 and −1 and a −log10p of 1.3 (*P* < 0.05).

Through comparing FTD-*GRN* samples against FTD-*MAPT* and FTD-*C9orf72* samples, 35 differentially expressed miRNAs were identified (26 upregulated versus 9 downregulated) (Fig. 5A). The top 5 upregulated miRNAs identified were miR-6874-3p, miR-362-3p, miR-21-3p, miR-1197 and miR-3615, whereas the top 5 downregulated miRNA cargoes were miR-454-3p, miR-135a-2-3p, let-7f-5p, miR-374a-5p and miR-184. Similarly, when comparing FTD-*MAPT* BDsEV samples against FTD-*GRN* and FTD-*C9orf72* samples, 38 miRNAs were also recognized (21 upregulated against 17 downregulated). Here, the top 5 upregulated miRNAs identified were miR-3129-5p, miR-1-3p, miR-449a, miR-1249-3p and miR-331-3p, where the top 5 downregulated miRNAs were miR-21-3p, miR-1197, miR-1287-5p, miR-3085-3p, miR-1286 (Fig. 5B). Lastly, through comparing FTD-*C9orf72* microenvironments against FTD-*GRN* and FTD-*MAPT* cargoes, only 1 upregulated miRNA was shown (miR-193b-5p), yet 15 downregulated miRNAs were identified (Fig. 5C). The top 5 downregulated miRNAs include miR-145-5p, miR-331-3p, miR-1249-3p, miR-125a-5p and miR-1-3p.

## Discussion

Diagnosing the different subgroups of dementia is essential when it comes to identifying the correct (and specific) therapeutic program and care for those with dementia, with the main aim to see dementia cases decrease internationally. With newer AD therapeutics such as Donanemab and Lecanemab being introduced to global markets, much excitement surrounds these groundbreaking drugs as these show major advancements which may revolutionize how to treat other NDs in the future. However, various governing bodies, especially within the UK, still state that such treatments are still not viable treatment methods for AD due to clinical diagnostic methods detecting AD too late for patients to benefit from such treatments.^35,36^ As aforementioned, studies have been conducted to show the diagnostic potential of miRNAs, where in longitudinal studies, miRNA have shown power with 87% sensitivity and 77% specificity to predict AD.^22^ More recently, specific miRNA such as let-7g-5p and miR-21-5p, have been identified in cellular and sEV environments to correlate strongly with PD cases linked to the *LRRK2* mutation,^37^ demonstrating the potential differentiating power that miRNA signatures may have. Therefore, using BDsEVs as a source to acquire potential miRNA cargoes directly from the organ most impacted by dementia and thus finding differences within the miRNA signatures from sEVs between the diseases potentially opens avenues to explore newer diagnostic markers.

In the current study, in group comparisons, only the two CMA-associated miRNAs, miR-224-5p and miR-106a-3p, demonstrated significant group differences; whereas through pairwise comparisons, only the upregulation of miR-224-5p was found to be significantly upregulated in AD and FTD-*MAPT* (*P* adj <0.05) compared with no disease controls. In 2013, Alvarez-Erviti *et al*.^33^ displayed the roles of miR-224-5p and miR-106a-3p in inhibiting LAMP2A and Hsc70, respectively, in PD. Whilst the role of the BDsEV-derived miR-224-5p was beyond the scope of the current project, this increase in AD and FTD-*MAPT* could suggest a potential pathogenic-specific dysregulation of LAMP2A in tauopathies, causing the further build-up of aggregated tau. In addition to NDs, in diseases such as breast cancers, miR-224-5p has also been shown to inhibit SMAD4, a known upstream activator of MA,^38^ suggesting a larger regulator role of miR-224-5p in proteostatic mediation of disease. Whilst pairwise comparisons of miR-106a-3p after Holm correction were not found (*P* adj > 0.05), unadjusted significant differences were observed through a significant upregulation in AD compared with no disease control and all FTD mutations. As stated, Alvarez-Erviti *et al*. (2013)^30^ showed the role of miR-106a-3p in PD in targeting Hsc70, suggesting increases of miR-106a-3p may lead to for Hsc70 dysregulation specifically in AD, driving for proteostatic imbalances—again, further experimentation is needed here. With respect to biomarkers, both miR-224 and miR-106a could be potential candidates for further biomarker validation experiments within peripheral biofluid sEVs, especially for tauopathies, yet functional assays should be performed to implement another layer of biological relevancy.

Further to this, no MA-associated miRNA showed group differences (*P* > 0.05), or survived pairwise comparisons after Holm corrections. Whilst miR-124 has shown to mediate MA through regulating AMPK and mTOR levels in dopaminergic neuronal cell models, no significant differences in pairwise comparisons were shown.^31^ However, miR-30a-5p was significantly downregulated in FTD-*GRN* and FTD-*C9orf72* compared with no disease control samples (*P* < 0.05) and miR-128-3p was significantly upregulated in FTD-*C9orf72* when compared with FTD-*GRN* (*P* < 0.05). Whilst many studies identify miR-30a as a mediator of autophagic regulation through inhibition of BCl-1,^39^ the downregulation in members of the miR-30 family (miR-30a, miR-30c and miR-30e) in macrophages leads to increases in Notch1 signalling, ultimately to low-grade inflammation.^32^ Further to this miRNA, miR-128-3p has been shown to regulate autophagy through various effects, such as deactivating JAK2/STAT3 signalling and TFEB-mediated MA in PD dopaminergic.^33,34^ Biologically, this may indicate autophagic-mediating pathways are dysregulated in FTD-*C9orf72* in comparison to FTD-*GRN*, though further testing of biological replicates and functional assays is necessary again to show biological relevance. With further biological repeats and experiments in biofluid sEVs, miR-30a and miR-128 could become potential candidates for the differentiation of TDP43-proteinopathies from other dementia diseases, alongside differentiation between each other.

Beyond candidate signals, we asked whether broader miRNA signatures exist. Using an exploratory approach to interrogate disease, subtype and mutation-specific miRNA signatures within BDsEVs, we performed three differential expression analyses (disease versus control; AD versus FTD; mutation versus mutation). However, when observing global microenvironments, no miRNAs survived FDR correction in any comparative analysis, therefore miRNAs that had significant nominal *P* values (*P* < 0.05) were shown. When identifying disease-specific (disease versus control) comparisons, distinct miRNA signatures were observed across multiple dementia subgroups, rather than single miRNA indicators. For example, the panel indicative of AD identified using small RNA-seq were upregulated miR-590-5p, miR-26a-2-3p, miR-6868-3p and miR-150-5p and downregulated miR-2681-5p, miR-1250-5p and miR-136-3p. These were distinctly different to those of the FTD mutations when compared with control samples, where even further miRNA was identified when comparing AD and FTD mutations directly. Interestingly, miR-26a-2-3p was not only upregulated in AD when compared with control, but also each FTD mutation too. This observation also shown by Leidinger *et al*.^40^ where miR-26a was upregulated in AD brain samples, suggesting that BDsEV miRNA cargoes reflect disease-relevant molecular changes occurring within the brain. In contrast, miR-26a-2 has been shown to be downregulated in PD brain samples,^41^ demonstrating further neurodegenerative-differentiating power potential due to high fold changes within AD samples.

When running pathway enrichment analysis on differentially expressed BDsEV miRNA, several KEGG pathways were consistent with impacted mechanisms of neurodegeneration (stress–response signalling pathways, cell cycle and senescence pathways, endocytic protein processing pathways) (Supplementary Data 5; Supplementary Fig. 4). The top pathways for both AD and FTD-*GRN* samples were colorectal cancer-linked pathways, where enrichment results that include cancer and other infection-associated pathways likely reflect shared mechanisms (including stress signalling, cell cycle and adhesion), rather than implying malignancy or infection within the cohort. For AD, the second top pathway highlighted was the immunoglobulin superfamily cell adhesion molecules (IgSF CAM) signalling pathway, which appears in all other sample results too at different ranks. This pathway is heavily involved in neurodevelopment through facilitating cell–cell interactions, was shown to be enriched.^42,43^ Members of this family, such as ICAM-1 and *N*-CAM, have been shown to promote neurotoxic actions such as neuroinflammation and vascular restructuring in multiple neurological disorders including HD, PD and vascular dementia.^44-46^ Specifically in AD, the APP is itself a cell adhesion molecule, where the interaction with other IgCAMs may promote or inhibit the production of Aß monomers, suggesting a close mediatory relationship with this pathway.^47,48^ For both FTD-*MAPT* and FTD-*C9orf72*, the top enriched KEGG terms converged on pathways of neurodegeneration, cell cycle and endocytosis, where each impact cell survival, pathogenic protein deliverance and endocytic dysfunction lead to downstream pathogenicity in NDs through the spreading of neuropathogenic cargoes and arresting cell development.^49,50^ Furthermore, in all NDs, other neuroprotective cellular stress responses, such as MAPK, ErbB and HIF-1 signalling were also flagged as enriched in the analysis, where the impairment of such pathways have been implicated.^51-53^ Given that BDsEVs may reflect the landscapes of both neuronal and glial cells, these findings suggest that altered sEV miRNA cargoes in dementia-derived EVs may contribute to intercellular signalling processes relevant to neurodegeneration and ageing.^54^

Here, we investigated BDsEV microRNA cargoes of AD and the three main genetic mutations of FTD to explore potential disease and mutation-specific biomarker signatures. Whilst brain tissues cannot be taken from patients, furthering this exploratory study may establish biologically and clinically meaningful small-transcriptomic biomarkers due to being derived directly from the source of impact. Through a literature search, whilst the prevalence of miRNA biomarkers of AD is a well investigated area, their role as potential markers of FTD (and causal mutations) are not as widely published suggesting a key area of expanse. Equally, as FTD is considered a spectrum of disorders which encompasses ALS, they also share key mutations such as *C9orf72*, *TARDBP*, *SQSTM1*, *FUS* and *TBK1,*^55^ suggesting that through further investigation, diseases such as these could also be differentiated. What’s more, NDs are complex, system-managed disorders where it is rare to find one specific dysfunctional unit in a ND-associated network that is compromised. In addition, single miRNAs are context-dependent and pleiotropic, therefore using a combination of miRNAs that shows a landscape rather than an event is more biologically informative, especially when differentiating diseases that are clinically similar.

In the current study, limitations have been identified that need to be considered when interpreting the results. Firstly, the brain is the third largest organ in the body, with four different lobes and many different regions within each lobe, all with very different and complex functions, therefore inherently heterogenous in nature due to regional complexity. Secondly, due to the nature of the FTD-*C9orf72* mutation, as different mutations of the *C9orf72* gene involves different numbers of the G_4_C_2_ repeat expansion, causing further variability within this mutation. To add another layer of complexity, the inter-individual differences (covariates) between people (age, race, ethnicity, gender and other demographics), also introduce higher levels of variability between post-mortem samples are introduced. When testing differences of age between diagnostic groups, significance was shown between the no disease control group and all disease groups. Whilst age is a massive factor in neurodegenerative diseases, age also impacts EV biology and protein expression also, which further suggests age could play a potential cofounding factor in this study. Due to the exploratory nature of this study, the small sample sizes of each group used and tissue availability, covariate adjustments were not realistic. Further studies involving brain tissue and peripheral fluids with larger, age-matched cohorts will be required to justify findings. Through running PCA (Supplementary Fig. 5), PC1 and PC2 accounted for 24% and 17% respectively, thus no complete diagnostic separation was observed. Ultimately, the dispersion across groups reflects the molecular heterogeneity of the human brain, displaying this expected inter-individual variability.

As stated, five post-mortem brain tissue samples per group were provided by the Manchester brain bank, yet due to costs associated with downstream molecular analyses (RT-qPCR and sequencing), subsets of 3 samples per group were selected. Whilst each subset was taken from the same diagnostic group and therefore treated as representative biological replicates of that group, these were not identical across assays used in the study. In addition, both RT-qPCR and small RNA-seq analyses involve testing multiple miRNAs simultaneously, requiring statistical corrections to control for false-positive findings. In studies such as this where biological replicates are limited, the use of conservative correction methods such as Holm and FDR adjustments can reduce the ability to detect subtle but biologically meaningful differences. Increasing cohort sizes of each group (control, AD, FTD mutation), whilst matching each demographical categories listen prior would improve statistical power, minimize variability and improve statistical estimates. Whilst the application of statistical corrections improves stringency and supports robustness of the strongest miRNA signal, such as miR-224-5p, an independent validation (in larger independent cohort or peripheral fluid sEVs) in a separate cohort is required to confirm reproducibility. Additionally, whilst this study focuses on BDsEVs isolated from the frontal lobe of the brain, incorporating other relevant areas of the brain, such as the temporal lobe, could broaden the study further and may highlight further differences and similarities seen.

Whilst this study isolated sEVs directly from the brain, further brain cell-specific markers for characterization, including NCAM, TMEM119 and CD44, could illuminate whether the sEVs were neuronal, microglial and astrocytic in nature respectively.^56-58^ Another issue is that due to the use of collagenase digestion coupled with column-based isolation, non-vesicular material such as myelin debris and protein aggregates may have been co-isolated with the BDsEVs. Further immunoblotting of myelin protein markers, such as PLP1 and MOG,^59^ and protein aggregate markers, including ubiquitin and HSP90,^60^ could increase the confidence when assessing whether preparations are disproportionately enriched for insoluble or aggregate-associated proteins. Together with other characterization limitations, an issue arises in terms of the post-mortem delay, the handling, recovery and storage of brain tissue. Due to these concerns, certain aspects may be impacted: sEV dynamics, such as morphology and cargo loading; miRNA expression profiles may differ; internal protein condition; and overall tissue condition.^61^ Like other limitations, this can also be rectified through increasing cohort size as any variation between samples caused by post-mortem delay will be minimized.

For RT-qPCR, were used SYBR green, which may offer lower sequence specificity than probe-based assays, especially within potential low-input sEV samples, thus findings should be interpreted in the context of methodological constraint. The normalization of RT-qPCR was performed using a cel-mir-39 spike-in control with equal miRNA input per reaction. However, this approach may not account for EV yield or particle number differences between samples, nor an endogenous miRNA reference. Future validation studies should therefore incorporate a potential endogenous miRNA control for the normalization of EV input alongside spike-in controls to strengthen comparisons. Lastly, whilst there is a lack of concordance between RT-qPCR and small RNA-seq datasets, this may reflect both methodological differences and the exploratory nature of the sequencing analysis, particularly in the context of low-input EV miRNA.

Using RT-qPCR and small RNA-seq, this study explored BDsEV miRNA cargoes and identified disease-specific and potentially disease-differentiating miRNA signatures of AD and genetic mutations of FTD. These findings highlight the utility of BDsEV miRNA as a foundation of discovery, to provide candidate markers for further investigations within peripheral biofluids such as CSF and blood, even saliva and urine. Using more accessible sources of sEVs, such as peripheral fluids within future studies could not only solidify findings but serve as a more available medium within clinical settings. Validation in independent cohort and sEVs from biofluids may support the development of faster, more precise and cheaper diagnostic methods for ND differentiation.

## Supplementary Material

fcag277_Supplementary_Data

## Data Availability

The data supporting the findings of the current study are available from the corresponding author (G.L.) upon reasonable request. Additionally, code is available from the corresponding author upon reasonable request.
