# Supplementary material for: Distinct brain extracellular vesicle microRNA profiles differ in frontotemporal dementia and Alzheimer’s disease
Source: Brain Commun. 2026 Jul 21;8(4):fcag277. doi: 10.1093/braincomms/fcag277 (PMC13435624; doi:10.1093/braincomms/fcag277)
Supplement: Supplementary file 1 [file fcag277_supplementary_data.zip › Supplementary_material.pdf]

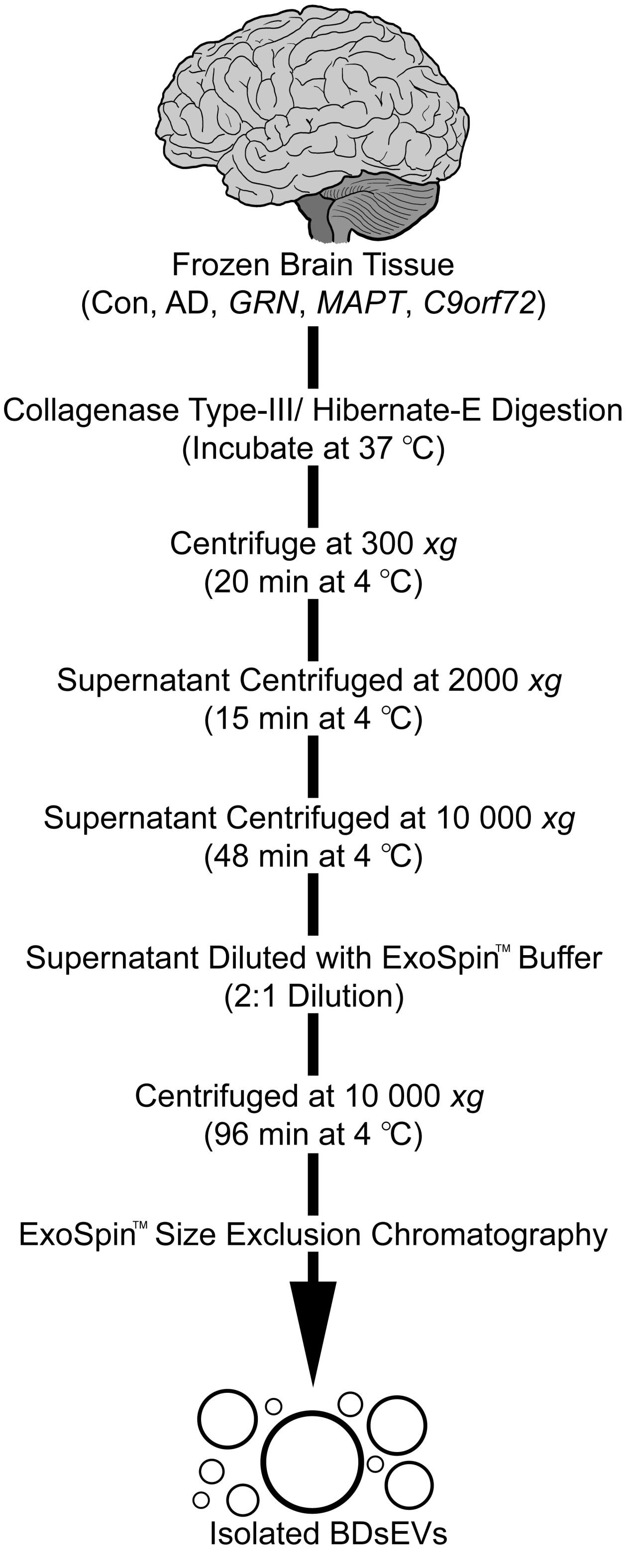

Supplementary Figure 1. Visualised workflow summarising the isolation of sEVs from fresh-frozen post-mortem brain tissue. Workflow shows serial high-speed centrifugation steps, couples with ExoSpin™ SEC isolation[S1]. Abbreviations: BDsEVs = brain-derived small extracellular vesicles.

Supplementary Methodology 1 – Characterisation of Brain-Derived Small Extracellular Vesicles using Three MISEV-Approved Approaches

For NTA, particle size and concentration were measured in light-scatter (NTA) and fluorescent (fNTA) modes on the ZetaView Nanoparticle Analyser (ZetaView Software version 8.06.01). Samples were diluted 1:1000 in PBS for NTA and read at 520 nm. For fNTA, to analyse the size and concentration of all biological particles, samples were stained with cell mask orange (CMO) dye at a dilution factor of 1:10,000,000 accomplished by a serial dilution: firstly 1:1000 CMO in PBS, then 1:10 with the sEV sample and finally 1:1000 in PBS again. Secondly, to analyse what percentage of biological particles beared characteristic CD markers, PE/Dazzle anti-human CD9, CD63 and CD81 markers (BioLegend) were diluted 1:100 in PBS, then 1:10 in the sEV sample (incubated for 2 hours), and 1:100 in PBS (final 1:100 000) and analysed at 550 nm. Particle concentration (particles/ml) were extracted per size plot for each biological replicate. To summarise particle burden across defined size ranges, area under curve (AUC) values were calculated per replicate using trapezoid numerical integration across four size windows: characteristic size (30-200 nm), mid-range (150-300 nm), large particles (300-900 nm) and full measured range (2.5-902.5 nm).

For characterisation with western blotting, BDsEV samples were compared against brain homogenates (BH) to reaffirm the absence of negative markers within BDsEV samples. For western blotting, 20 µg EV and BH protein was prepared in 4x loading buffer (EVs heated at 70 °C, BH at 95 °C) and run on 10% SDS-PAGE gels (in 1X Tris-Glycine-SDS) at 100 V before being transferred to nitrocellulose membranes (in 1X Tris-Glycine-MeOH). Membranes were blocked in 5% BSA/ Milk for 1 h, incubated with primary antibodies overnight at 4 °C, and secondary anti-mouse-HRP for 2 h (concentrations and dilutions in Supplementary Table 1), then developed with SuperSignal™ West Atto

Ultimate Sensitivity Chemiluminescent Substrate solution and imaged on a LI-COR Odyssey Fc Imager.

Supplementary Table 1. Concentrations and dilutions of antibodies used to characterise BDsEVs for western blot analysis.

Characterisation Antibody	Size (kDa)	Company	Final Concentration (µg/ml)	Dilution Factor
Primary mouse anti-human GM130 Monoclonal Antibody (6662-1-Ig)	130	Proteintech	0.2	1:5000
Primary mouse anti-APP Monoclonal Antibody (60342-1-Ig)	100	Proteintech	1	1:1000
Primary mouse anti-human Calnexin Monoclonal Antibody (66903-1-Ig)	90	Proteintech	0.1	1:10000
Primary mouse anti-human CD63 Monoclonal Antibody (10628D)	40-60	Invitrogen	0.25	1:2000
Primary mouse anti-human Flotillin 1 Monoclonal Antibody (67968-1-Ig)	47	Proteintech	0.5	1:2000
Primary mouse anti-human CD81 Monoclonal Antibody (10630D)	25	Invitrogen	0.25	1:2000
Primary mouse anti-human CD9 Monoclonal Antibody (10626D)	25	Invitrogen	0.25	1:2000
Secondary goat anti-mouse IgG, HRP-linked Antibody (#7076)	-	Cell Signalling Technology	-	1:5000

Transmission electron microscopy (TEM) was used to provide another dimension of size and morphology characterisation of sEVs. Samples were sent to the University of Liverpool to be processed and imaged using TEM. Here, 10 µl of sEV sample was fixed in 1% glutaraldehyde, washed with ddH_2O, stained with 1% uranyl acetate, and incubated on 4% uranyl acetate/ 2% methyl cellulose (1:9) at 4°C for 10 mins. Samples were dehydrated through graded alcohols and imaged using FEI 120 kV Tecnai G2 Spirit BioTwin transmission electron microscope.

Supplementary Table 2. Forward primer sequences designed for RT-qPCR experiments. Using miRbase (https://www.mirbase.org/), forward primers were designed and ordered from Eurofins Scientific, for use within the MasterMix. Alongside the sequence of the miRNA, GC content (%), melting point (°C) and molecular weight (g/mol) of each primer is provided.

MicroRNA	Sequence (5' – 3')	GC (%)	Tm (°C)	MW (g/mol)
hsa-miR-124-3p	UAAGGCACGCGGUGAAUGCCAA	55	62.1	6793.5
hsa-miR-30a-3p	UGUAAACAUCCUCGACUGGAAG	45	58.4	6743.4
hsa-miR-128-3p	UCACAGUGAACCGGUCUCUUU	48	57.9	6372.1
hsa-miR-224-5p	UCAAGUCACUAGUGGUUCCGUUUAG	44	61.3	7663.0
hsa-miR-373-5p	ACUCAAAAUGGGGGCGCUUUCC	55	62.1	6735.4
hsa-miR-106a-3p	CUGCAAUGUAAGCACUUCUUAC	41	56.5	6669.3
hsa-miR-26b-5p	UUCAAGUAAUUCAGGAUAGGU	33	52.0	6484.3
cel-miR-39	UCACCGGGUGUAAAUCAGCUUG	50	60.3	7049.1

Supplementary Table 3. RT-qPCR cycling conditions.

Step	Temperature (°C)	Time (s)
Denaturation	95	30
Annealing and Extension	60	60
Cycle Repeated for 40 Cycles		

Supplementary Methodology 2 – Small RNA-Sequencing Library Preparation

Each library was prepared using the small RNA-seq library preparation kit (Lexogen). Prior to library preparation, the 3’- and 5’- adapter, and reverse transcription primer were diluted by 0.5x to correct for lower RNA amounts (<100 ng). To start, 6 µl 10 ng RNA from each diagnostic group BDsEV were mixed with 1 µg 3’ adapter (5’-TGGAATTCTCGGGTGCCAAGGAACTCCAGTCAC-3’) and incubated for 2 min at 70 °C to denature and resolve secondary structures before ligation. The first MasterMix of 12 µl ligation mix and 1 µl enzyme mix) was added to the denatured RNA/ 3’adapter sample and incubated for 1 h at 28 °C to complete 3’ adapter ligation. A purification step was performed where each reaction was added to a purification column, washed with 100% ethanol and column buffer. For 5’ adapter ligation, the 5’ adapter was denatured by incubating for 2 min at 70 °C and was added to MasterMix 2 (1 µl denatured 5’ adapter, 11 µl ligation mic and 1 µl enzyme mix) which in turn, 13 µl was added to each reaction. This mixture was incubated for 1 h at 28 °C to complete 5’ adapter ligation. The reaction RNA mixture with 3’ and 5’ adapters was then converted to cDNA using 1 µl reverse transcription primer. Each mixture was incubated for 2 min at 70 °C, where 9 µl MasterMix 3 (8 µl first strand cDNA synthesis mix and 1 µl enzyme mix) was added to each reaction. This was heated for 1 h at 50 °C, creating the RNA/ cDNA library ready for amplification.

For amplification, P5 and P7 (small RNA i7 primers with unique 6 nucleotide barcoding sequences) adapter sequences were added for cluster generation using endpoint PCR, creating copies of ID-tagged DNA. For each RNA/cDNA library, 64 µl endpoint PCR MasterMix (50 µl PCR mix, 3 µl P5 primer and 11 µl H_2O) was added alongside 3 µl unique Sri7 index primer, where each library underwent endpoint PCR. To purify, each reaction was added to a purification column, washed with 100% ethanol and column buffer,

eventually eluting to remove PCR elements that could interfere with downstream quality and quantification methods and sequencing itself.

Supplementary Table 4. Endpoint PCR cycling conditions.

Step	Temperature (°C)	Time
Initial Denaturation	98	30 s
22 Cycles		
Denaturation	98	10 s
Annealing	60	30 s
Extension	72	15 s
Final Extension	72	10 min

At this point, TapeStation High Sensitivity DNA 1000 (D1000) ScreenTape Assay (Agilent) was used as a quality control to observe average library fragment size and contaminants. For the quantification of each generated library, Qubit 1x dsDNA high sensitivity quantification kit (ThermoFisher) was used. For library multiplexing of four individual samples, both library fragment length (bp) and concentration (ng/ul) were normalised. Prior to denaturing, 5 µl 4 nM libraries were diluted to 4 nM with PCR-grade H_2O. Each diluted library was denatured through mixing with 5 µl 0.2 N NaOH, agitated and centrifuged at 280 *xg* for 1 min, where after each library was incubated for 5 min at room temperature. The denatured libraries were further diluted using 990 µl HT1, generating a 20 M library, again further diluted to 8 pM with HT1. For experiment, a PhiX control library was used as a spike-in standard for cluster generation, sequencing and alignment. The libraries were loaded onto an Illumina MiSeq v3 flow cell and sequenced using the Illumina MiSeq sequencer, where sequencing ran a 50-cycle read.

Supplementary Methodology 3 – Bioinformatic Workflow

For small RNA-Seq, quality assessment of each run was performed using FastQC (v0.12.1) to assess base quality, GC content, sequence length distribution and adapter contamination,

after which CutAdapt (v5.2) was used to remove the Illumina small RNA adapter sequence (TGGAATTCTCGGGTGCCAAGGACCTCCAGTCAC). Post-trim quality was reassessed again using FastQC, with all reads passing QC retained. To map short reads (around 18-30 nt), Bowtie2 (v2.5.4) was built from the hg38 human reference genome (downloaded from UCSC Genome Browser). Reads were aligned to hg38.fa reference using very sensitive mode and single-end, end-to-end alignment. Sam files were converted to sorted BAM files using samtools (v1.22.1) where alignment statistics were quantified using the flagstat function. For each sample, miRNAs were quantified using a GFF3 annotation from miRBase (https://www.mirbase.org/) with HTSeq-count (v2.0.9) where all samples were merged into a single count matrix for downstream analysis.

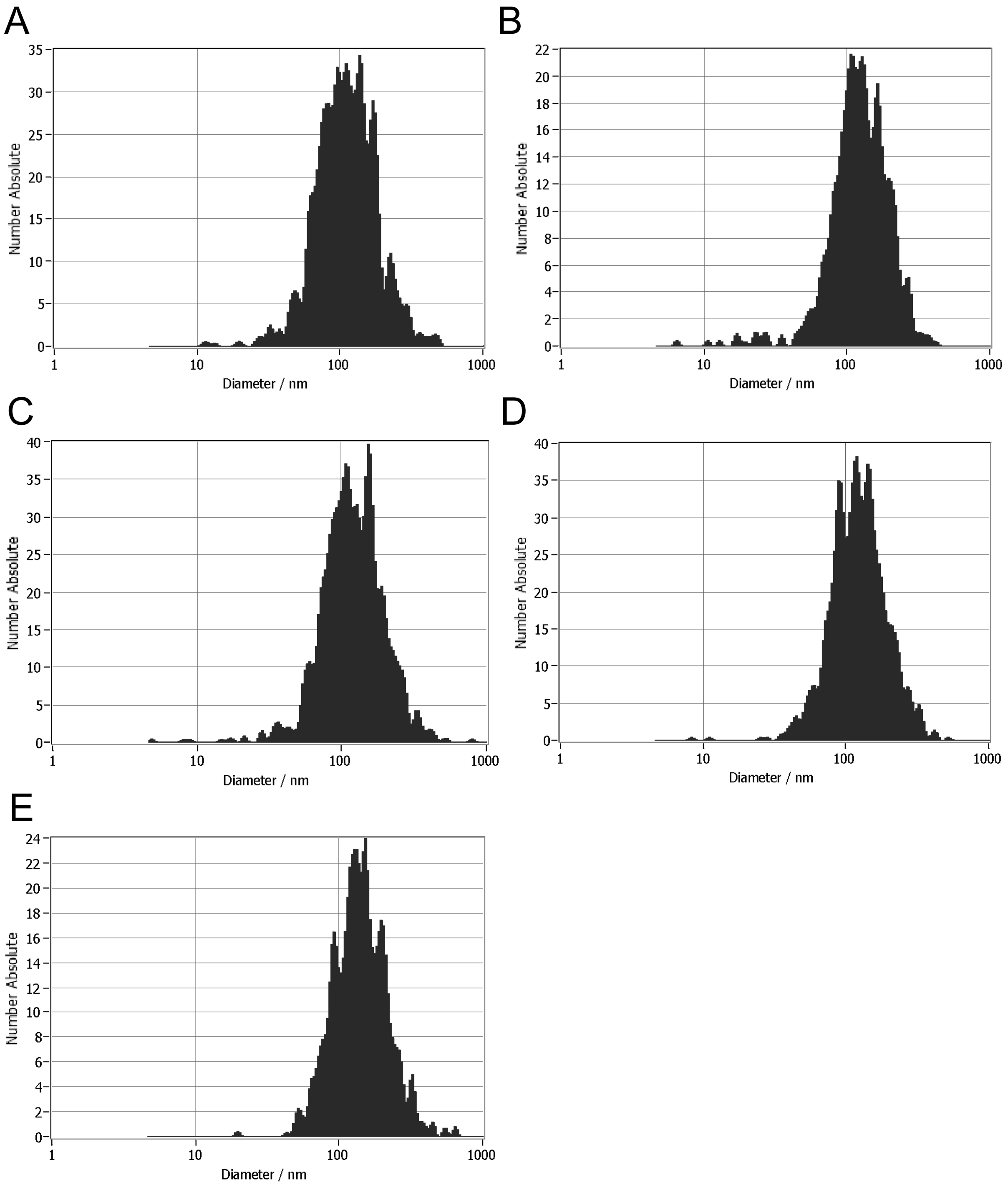

Supplementary Figure 2. Native plots (Absolute Particle Number against diameter (nm)) taken directly from the ZetaView output files. Shown are plots from one replicate of (A) control, (B) AD, (C) *GRN*, (D) *MAPT* and (E) *C9orf72*.

Supplementary Table 5. NTA run information including instrument parameters (version, sensitivity, shutter level, number of positions analysed, dilution factors of both CMO and CD markers, pH, temperatures and viscosities). From a starting tissue amount of 250 mg, isolated BDsEVs were measured using NTA where measurements were obtained using 11 positions, using fixed settings to fall within instrument's recommended particle concentration range. To further ensure quality, the cell was washed twice between samples using wash buffer.

**Instrument Settings**				
**Version**	8.06.01			
**Camera Level**	**Sensitivity**	96	**Shutter**	100
**Gain**	44.16			
**Brightness**	**Min**	30	**Max**	255
**Area**	**Min**	10	**Max**	1000
**No. of Positions**	11			
**sEV Dilution Factor (in CMO)**	1:10 000			
**sEV Dilution Factor (in CD)**	1:1000			
**pH**	7.0			
**Temperature**	**Replicate 1**	**Replicate 2**	**Replicate 3**	**Average**
Control	28.94	25.81	29.14	27.96
AD	28.35	28.02	28.52	28.30
GRN	28.93	29.07	29.21	29.07
MAPT	29.44	29.42	29.52	29.46
C9orf72	29.11	29.21	29.37	29.23
**Viscosity**	**Replicate 1**	**Replicate 2**	**Replicate 3**	**Average**
Control	0.883302	0.819356	0.815270	0.839309
AD	0.831409	0.838151	0.827936	0.832499
GRN	0.819560	0.816700	0.813840	0.816700
MAPT	0.809141	0.809549	0.807506	0.808732
C9orf72	0.815883	0.813840	0.810571	0.813431
**Average CMO Particle Count**	**Replicate 1**	**Replicate 2**	**Replicate 3**	**Average**
Control	4.35×10^5	3.46×10^6	3.91×10^6	2.60×10^6
AD	3.23×10^6	3.04×10^6	3.19×10^6	3.15×10^6
GRN	3.76×10^6	4.00×10^6	4.27×10^6	4.01×10^6
MAPT	2.94×10^6	3.08×10^6	3.05×10^6	3.02×10^6
C9orf72	1.36×10^6	1.27×10^6	1.24×10^6	1.29×10^6

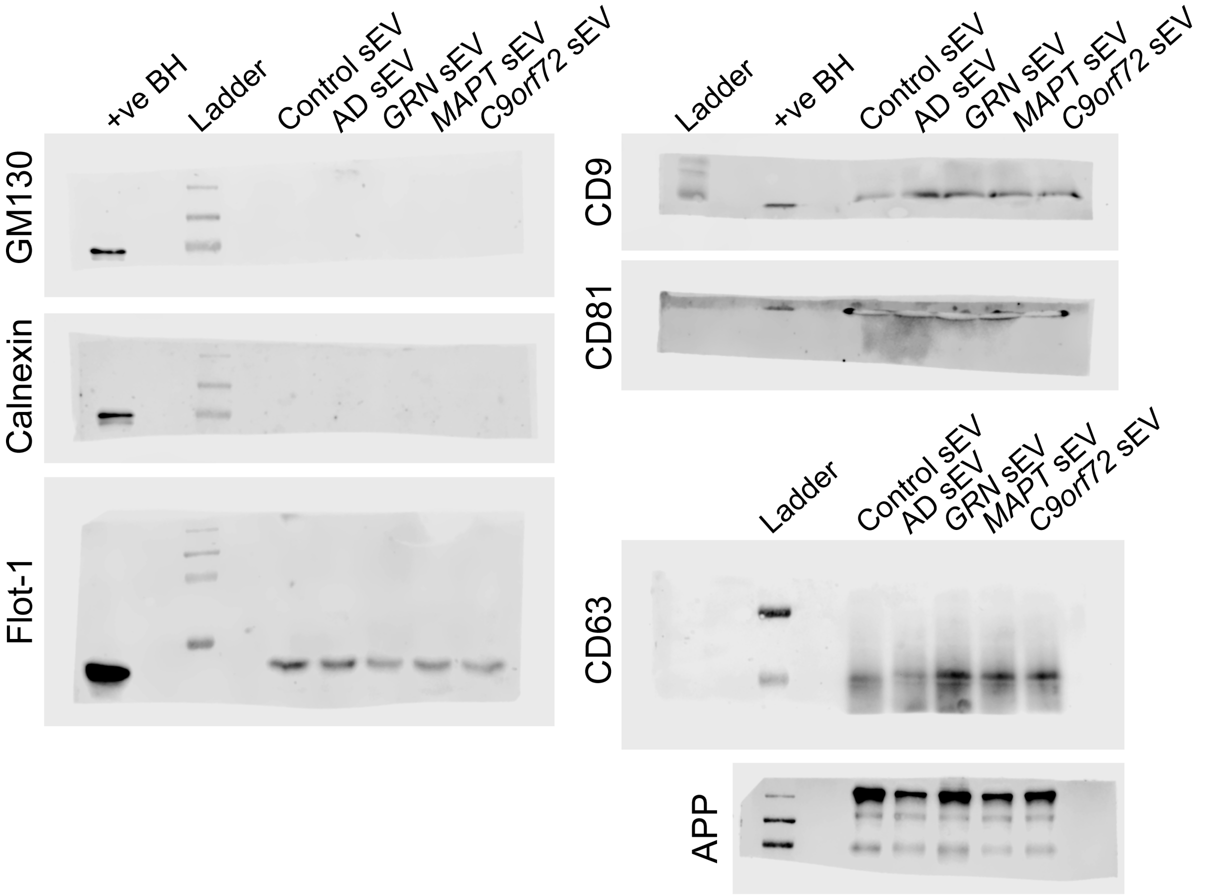

Supplementary Figure 3. Full western blot images of both negative and positive characterisation markers of BDsEVs. The presence of the negative GM130 and Calnexin is shown within the whole BH sample yet absent within the isolated BDsEV samples in all diagnostic group samples. The presence of Flot-1, CD9, CD81 and CD63 and APP are displayed in both BH and BDsEVs samples. All except CD63 and APP and are shown ran against a positive BH sample. Furthermore, the images shown were trimmed as to probe for more than one antibody at a time.

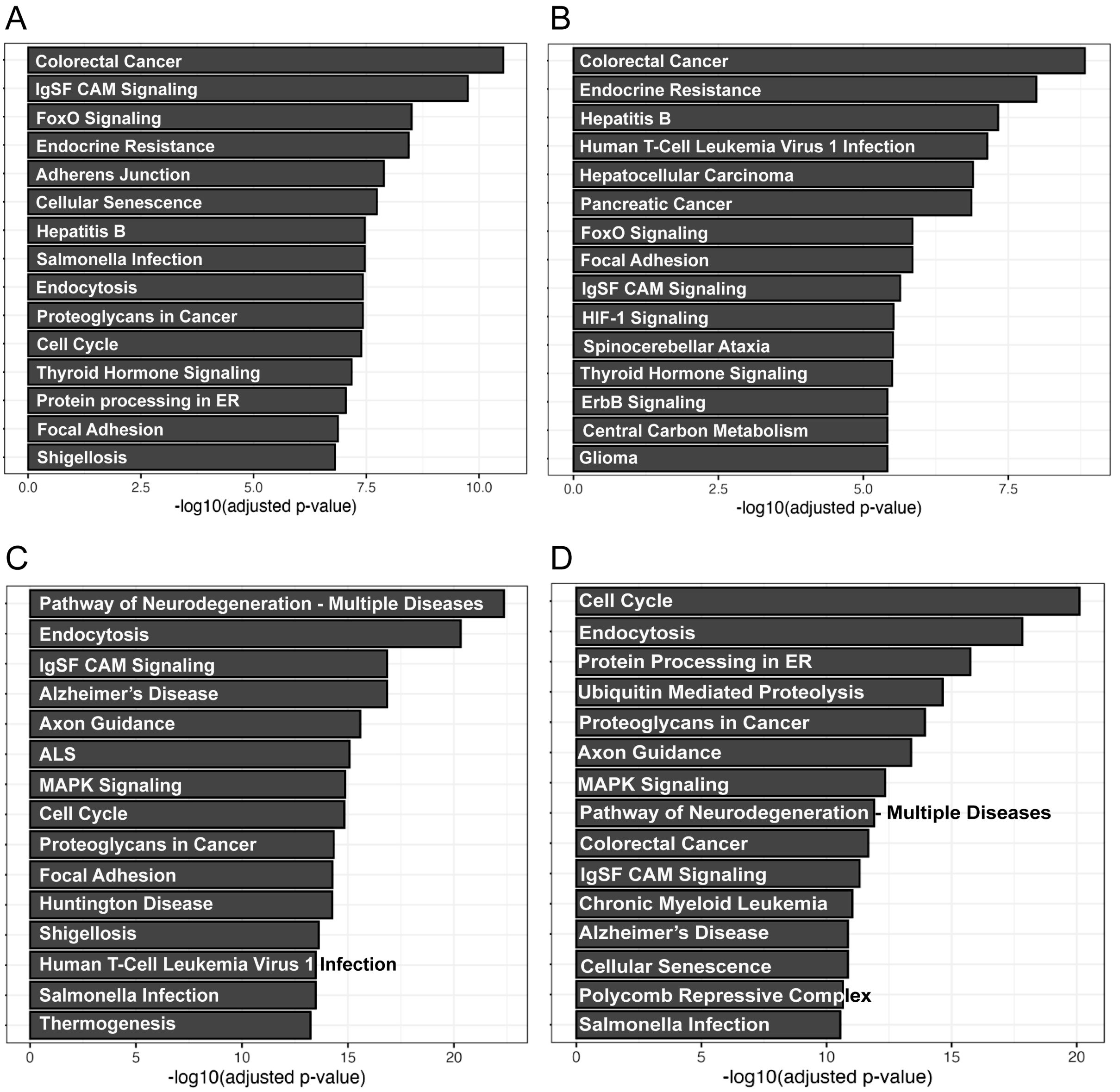

Supplementary Figure 4. KEGG pathway enrichment analysis of differentially expressed miRNAs across diagnostic groups. Pathway enrichment analysis of predicted gene targets of differentially expressed miRNAs of (A) AD vs control (B) *GRN* vs control, (C) *MAPT* vs control and (D) *C9orf72* vs control, ranked by -log10(Benjamini-Hochberg (FDR) adjusted p value <0.05). Abbreviations: ALS – amyotrophic lateral sclerosis.

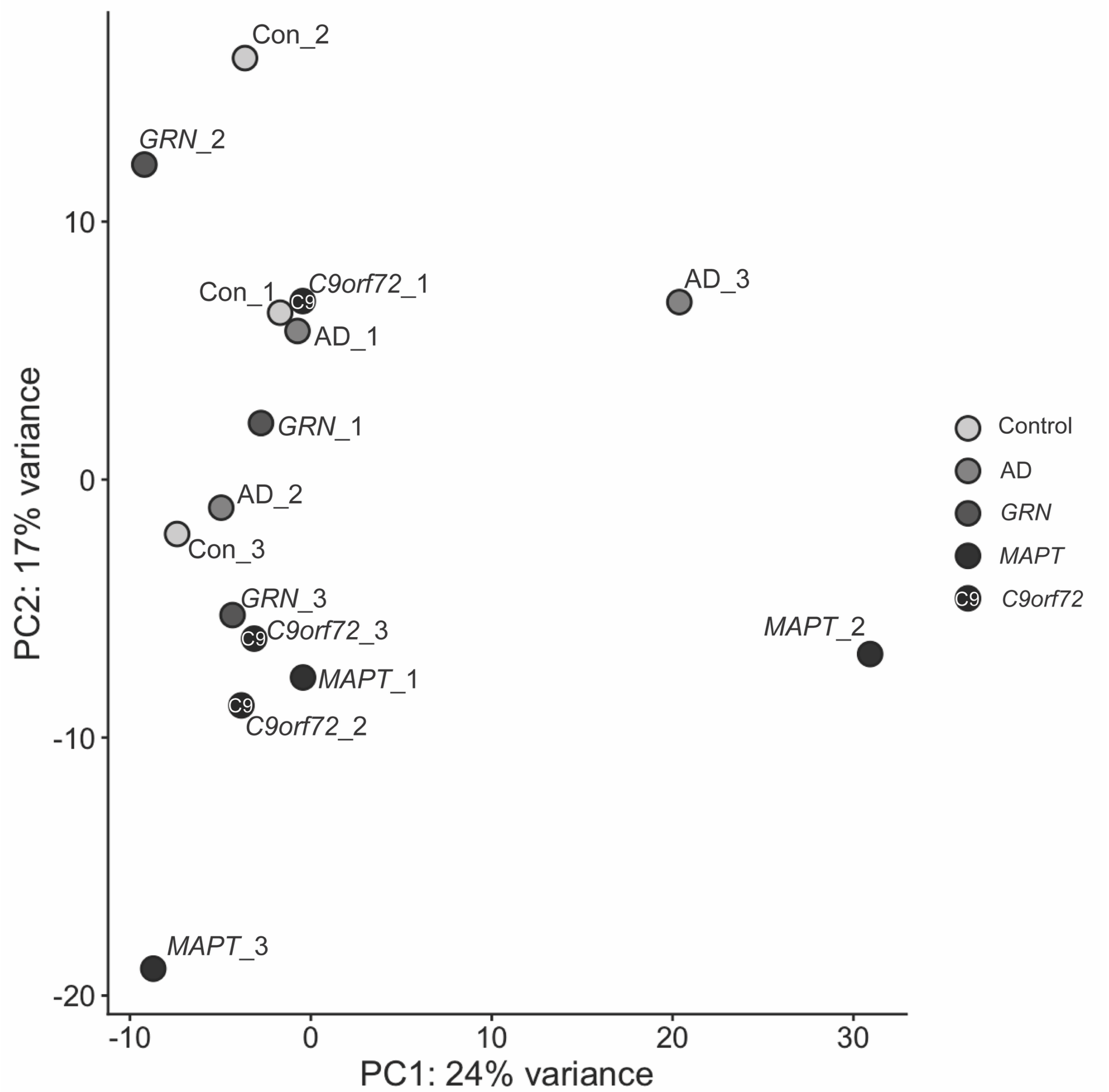

Supplementary Figure 5. PCA of variance-stabilised miRNA expression profiles from BDsEV samples across diagnostic groups. PCA was used as an exploratory analysis to assess global expression variance, where labelled points represent individual samples within each group.

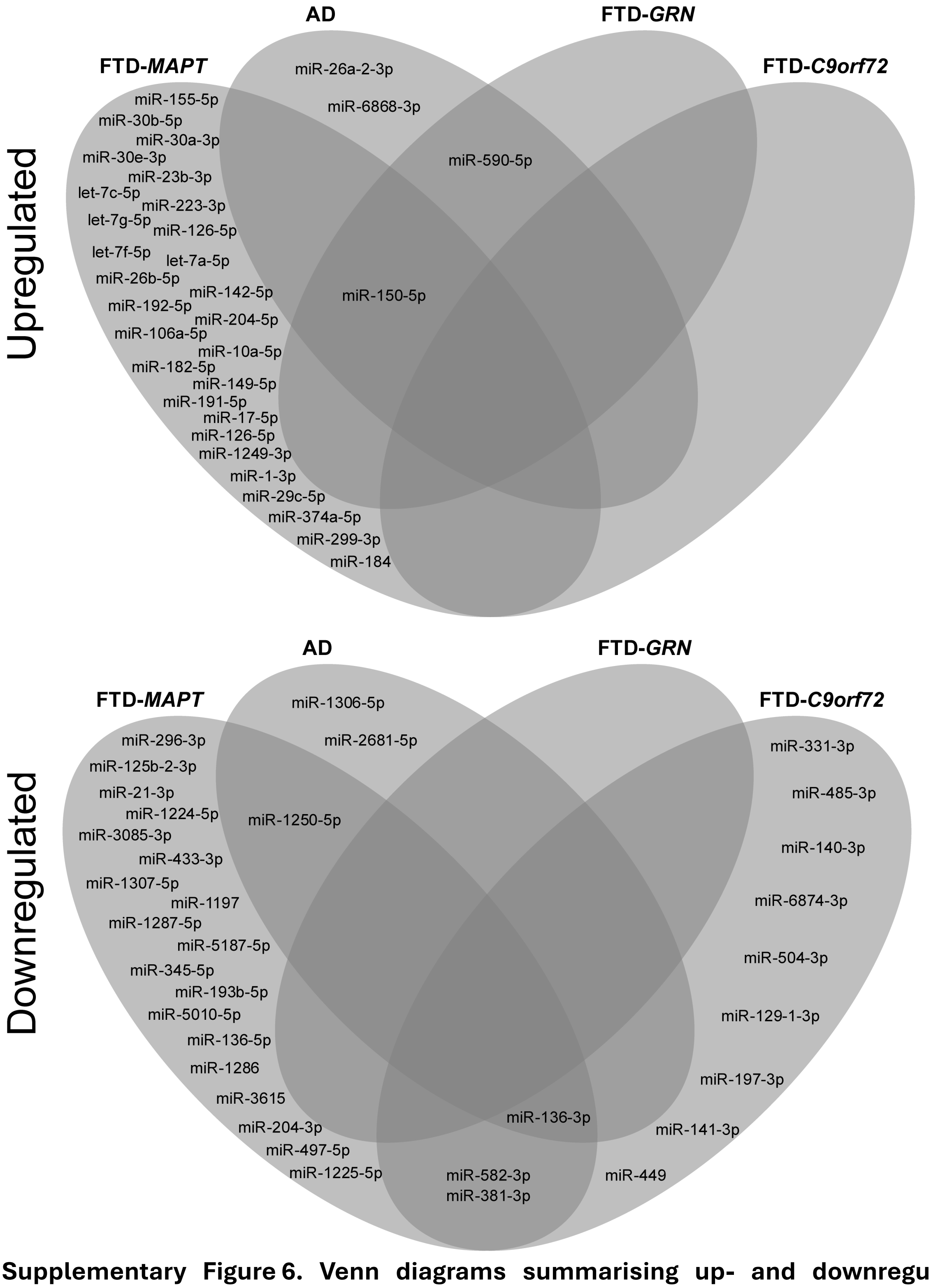

Supplementary Figure 6. Venn diagrams summarising up- and downregulated individual and shared miRNAs of each disease subtype compared to no disease control samples.

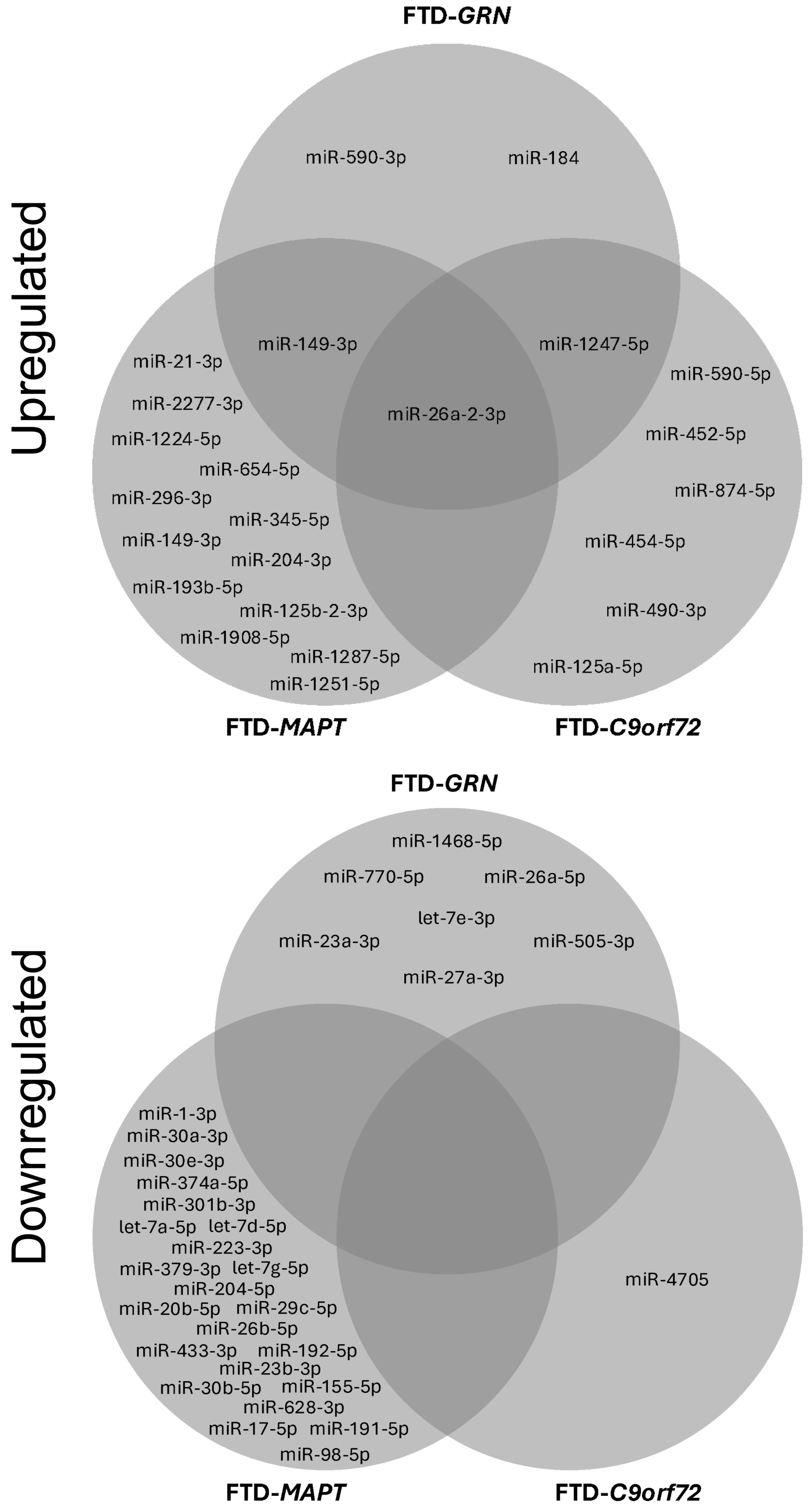

Supplementary Figure 7. Venn diagrams summarising up- and downregulated individual and shared miRNAs of each FTD mutation compared to AD samples.

Supplementary Reference

S1. Wang T, Anderson KW, Turko IV. Assessment of Extracellular Vesicles Purity Using Proteomic Standards. *Analytical Chemistry*. 2017;89(20):1-13.
